# When Two Species Meet: A Potential Beetle‐Yeast Facultative Mutualism

**DOI:** 10.1111/1758-2229.70157

**Published:** 2025-07-14

**Authors:** Rodolfo Bizarria, Tatiane de Castro Pietrobon, Pepijn W. Kooij, Andre Rodrigues

**Affiliations:** ^1^ São Paulo State University (UNESP), institute of Biosciences Rio Claro, São Paulo Brazil

**Keywords:** endophytic yeast, *Fabaceae*, non‐obligate mutualisms, *Phaffomycetaceae*, *Scolytini*, volatile compounds

## Abstract

Facultative mutualisms, such as some insect‐yeast interactions, can be established between partners that only interact in certain stages of their life cycles. While exploring yeasts associated with Brazilian beetles, we found a particular *Cyberlindnera* yeast associated with *Spermophthorus apuleiae*. This yeast was found within the beetles' galleries, which are excavated in fruits of *Libidibia ferrea*, a native Brazilian tree. We isolated a total of 15 yeast and yeast‐like species associated with the fruits and beetles, mostly from the orders *Phaffomycetales* and *Serinales*, and explored their role in yeast‐beetle interactions. *Cyberlindnera* is the most recurrent yeast, found in 90% of the fruit samples infested with beetles and representing 79% of the total isolates. Results from bioassays support the interaction between *Cyberlindnera* and *Spermophthorus apuleiae*. We hypothesise that reciprocal benefits may underlie this association: beetle gallery excavation boosts the density of *Cyberlindnera* in fruits, while the yeast produces attractive volatiles to beetles. These volatiles are usually associated with signalling towards sugar resources that enhance dispersal, an idea that warrants further exploration. We consider the *Cyberlindnera*‐beetle association a potential model for studying the ecology and evolution of non‐obligate (facultative) mutualisms.

## Graphical abstract

A *Cyberlidnera* yeast was discovered within galleries formed by *Spermophthorus apuleiae* beetles in the fruits of Ironwood trees. We hypothesise this association is a non‐obligate mutualism. Non‐obligatory as partners are not always found together, and mutualistic, as the beetles increase the yeast's density in the fruits, while the yeast produces volatile compounds that attract the beetles.

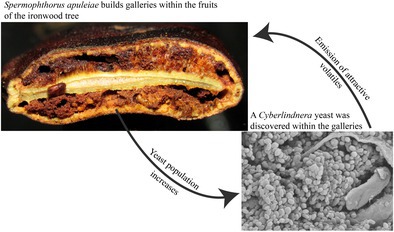

## 1 Introduction

Mutualisms, an ecological interaction in which both organisms benefit, play key roles in ecosystems (Asplund and Wardle 2017; Meyer et al. 2011), impacting species richness and diversity (Chomicki et al. 2019), and enhancing niche modification (Buser et al. 2014). In some mutualisms, the partners are not physically connected—meaning they are not in direct physical contact, attached to one another, or living inside each other—and their interactions occur only in specific stages of their life cycle (Boucher et al. 1982; Rafferty et al. 2015), as is the case of plant pollination and seed dispersal (Howe 1984; Rafferty et al. 2015), ant‐extrafloral nectary interactions (Keeler 1981) and anemone‐dwelling fishes (Boucher et al. 1982). Yeasts are often associated with insects, and their interactions are usually referred to as diffuse mutualisms (Starmer and Lachance 2011; Madden et al. 2018) since yeast communities interact with different species of insects. The *Drosophila*‐yeast mutualism is the most studied case (Starmer and Fogleman 1986; Buser et al. 2014), and is occasionally classified as diffuse mutualism (Starmer and Lachance 2011; Chakraborty et al. 2022). In this interaction, yeasts provide nourishment for the adults and their larvae (Bellutti et al. 2018; Starmer and Fogleman 1986), or perform environmental detoxification (Starmer and Fogleman 1986). In turn, the fly promotes the dispersal of yeasts through different environments (Gilbert 1980; Ganter 1988).

Yeast dispersal mediated by insects may occur externally, with yeasts attached on the insect integument (Arcuri et al. 2014), within specialised mycangial structures (Toki et al. 2012) or in the gut (Stefanini 2018). Insect guts can support yeast mating and hybridisation, favouring their dispersal and survival, as observed in *Drosophila* (Reuter et al. 2007) and social wasps (Stefanini et al. 2016). Yeast metabolites also provide chemical signals towards sugar resources, enhancing their dispersal by insects (Scheidler et al. 2015; Koerte et al. 2020). Beetles have previously been reported to facilitate yeast dispersal (Lachance et al. 2001), and the chemical attraction to yeast volatiles has been demonstrated in different species, including the frugivorous *Carpophilus* beetles (Baig et al. 2020) and the small hive beetle *Aethina tumida* (Torto et al. 2007; Benda et al. 2008). In some cases, even specialised organs (i.e., mycetangia, Vega and Biedermann 2020) can host yeast cells supporting their dispersal, as seen in stag beetles (Yamamoto and Toki 2023), the western pine beetle *Dendroctonus brevicomis* (Davis et al. 2011), the non‐social lizard beetle *Doubledaya bucculenta* (Toki et al. 2012), and the ship‐timber beetle *Elateroides dermestoides* (Toki 2023).

Among the benefits provided by yeasts to beetles, some species rely on yeasts for nourishment, as is the case of *Wickerhamomyces anomalus* (Toki et al. 2012), a nutrient source for larvae of *D. bucculenta* . Another potential benefit is the detoxification of harmful compounds in the environment, such as by *Cyberlindnera americana* associated with *Dendroctonus* spp. (Rivera et al. 2009; Lou et al. 2014; Briones‐Roblero et al. 2017); genome and transcriptome evidence suggests a role in detoxification (Soto‐Robles et al. 2019). In the mountain pine beetle *Dendroctonus ponderosae* , yeasts have been shown to produce anti‐aggregation pheromones (Hunt and Borden 1990). They can also release volatile compounds that may enhance or outcompete the growth of filamentous fungi associated with beetles, as in *D. brevicomis* (Davis et al. 2011) and *D. ponderosae* beetles (Adams et al. 2008). Since beetles and their environments are known to harbour different yeasts (Nguyen et al. 2006; Suh et al. 2005; Suh and Blackwell 2004, 2005), many interactions remain unexplored.

During a survey for yeasts associated with beetles, we found a high density of yeasts in fruits of *Libidibia ferrea* Mart. ex Tul. when infested by the borer beetle *Spermophthorus apuleiae* (Scolytinae, Curculionidae: Coleoptera). This beetle is known to have a frugivorous lifestyle, constructing galleries in the fruit mesocarp without harming the seeds (Suesdek and Lima 2011), and it is a common insect associated with this plant species (A. Costa Lima 1929). Fruits of this native tree in Brazil, popularly known as ‘Jucá’, ‘Pau‐ferro’, or Brazilian ironwood (Oliveira and Fernando 2024), are known to contain phenolic compounds (Ueda et al. 2001; Nakamura et al. 2002; Ferreira et al. 2016; Comandolli‐Wyrepkowski et al. 2017). However, the influence of fruit chemical composition on ecological interactions remains poorly understood. Moreover, despite the widespread distribution of *L. ferrea* in Brazil, few studies beyond taxonomic classification have explored the biology of *S. apuleiae*, its associated microbiota, and aspects of its behavioural ecology, such as the roles of males and females within galleries.

Although the diversity and ecological roles of yeasts associated with *S. apuleiae* remain largely unexplored, the high yeast density observed in infested fruits suggests yeasts may influence beetle fitness. To address this knowledge gap, we pose the following questions: What is the community structure of yeasts associated with beetle‐infested fruits? Do the yeasts produce volatiles that influence beetle behaviour? Here, we show that *S. apuleiae* beetles interact not only with *L. ferrea* but also with a yeast community inhabiting the galleries it excavates in the fruit mesocarp. We tested the hypothesis that yeasts are autochthonous to the fruits and confirmed that yeast abundance increases as a result of beetles' tunnelling activity. Our findings also indicate that yeasts with affinities to the genus *Cyberlindnera* are frequently found in this association and produce volatiles to which the beetles are attracted. These volatiles may contribute to yeast dispersal and could explain the observed specificity within this yeast‐insect interaction. Alternatively, the volatiles may be of a more general nature, with beetle attraction occurring stochastically rather than through specificity. Chemical characterisation of the yeast volatile emissions and their metabolic pathways will help test this alternative hypothesis. We also demonstrate that this short‐term interaction, though non‐obligate (facultative), involves a specific niche shared between the partners. Based on our results, we hypothesise that this is a case of mutualism, with yeast benefiting from beetle dispersal and through gallery excavation, while the beetle is guided by attractive by‐products produced by the yeast. Conversely, future investigations of beetle fitness may clarify this interaction as a case of symbiosis.

## 2 Experimental Procedures

### 2.1 Yeast Isolation

To assess the yeast community associated with fruits of *L. ferrea*, we sampled fruits infested by *S. apuleiae* and fruits without the beetle from a total of 10 different trees from three localities in Brazil (Rio Claro‐SP, Limeira‐SP, Mogi‐Guaçu‐SP, Table 1). All samples were aseptically placed into sterile plastic containers and transferred to the laboratory for yeast isolation. We surface‐disinfected fruits with 70% ethanol and opened them with sterile instruments. For yeast isolation from the fruits, we made an opening of approximately 2 cm^2^ in fruits, with or without the presence of beetles, and we scraped and suspended the mesocarp in PBS 1× (g L^−1^: 8 NaCl; 0.2 KCl; 1.44 Na_2_HPO_4_; 0.24 KH_2_PO_4_; pH = 7.4) supplemented with 0.05% Tween 80 (Sigma‐Aldrich, St. Louis, MO, USA). For yeast isolation from beetles, we sampled six males and six females from infested fruits of each of the 10 different trees from three localities in Brazil (Rio Claro‐SP, Limeira‐SP, Mogi‐Guaçu‐SP, Table 1). To isolate yeasts strongly adhered to the beetle exoskeleton, we pooled the beetles and washed them two times in PBS 1× supplemented with 0.05% Tween 80. To isolate internal yeasts, we disinfected beetles by immersion in 70% ethanol (30 s), washed them three times in sterile water, and then crushed them in 1 mL of PBS 1× supplemented with 0.05% Tween 80. We diluted this suspension to 1:100 and 1:1000, and 100 μL were surface‐spread on Yeast Extract–Peptone–Dextrose Agar (YPD: 2.0% dextrose; 2.0% peptone; 1.0% yeast extract and 1.5% agar) supplemented with 150 μg mL^−1^ of chloramphenicol (Sigma‐Aldrich, St. Louis, MO, USA) and with the pH adjusted to 4.5 (with 0.1 N HCl). We also aseptically removed the accumulated fruit material on the excavated front of males' heads and suspended it in PBS 1×, followed by inoculation in 25 mL YPD at 25°C under 120 rpm of rotation. To confirm the prevalence and distribution of yeast taxa in the beetle's galleries, we performed a second isolation attempt from infested fruits from 21 additional trees, distributed over eight different localities in the São Paulo state (Table 1).

**TABLE 1:** Sample identification and abundances (CFU mL^−1^) of recovered yeasts.

Sample ID	Coordinates (lat long)	City‐state	ME‐male^a^	ME‐female	MI‐male	MI‐female	Fruits^b^
RB191118‐01	S22°23′54.2″ W47°33′12.5″	Rio Claro‐SP	1.05E + 02	4.00E + 01	1.20E + 02	6.00E + 01	1.34E + 06
RB191118‐02	S22°23′47.2″ W47°32′49.0″	Rio Claro‐SP	0.00E + 00	1.00E + 01	9.50E + 02	5.00E + 01	3.60E + 05
RB191118‐03	S22°23′47.8″ W47°32′45.2″	Rio Claro‐SP	0.00E + 00	1.00E + 02	0.00E + 00	2.40E + 02	2.66E + 06
RB191118‐04	S22°23′46.6″ W47°32′45.1″	Rio Claro‐SP	0.00E + 00	7.00E + 01	0.00E + 00	4.00E + 01	8.80E + 05
RB191118‐05	S22°23′43.0″ W47°32′46.0″	Rio Claro‐SP	6.00E + 01	0.00E + 00	0.00E + 00	1.68E + 03	1.66E + 04
RB200606‐01	S22°33′21.9″ W47°24′46.8″	Limeira‐SP	1.00E + 01	0.00E + 00	0.00E + 00	0.00E + 00	1.03E + 05
RB200606‐02	S22°33′20.8″ W47°24′46.8″	Limeira‐SP	2.00E + 01	1.00E + 01	2.60E + 02	1.00E + 01	1.08E + 05
RB200606‐03	S22°33′21.0″ W47°24′45.6″	Limeira‐SP	0.00E + 00	7.00E + 01	2.01E + 03	4.00E + 01	2.51E + 05
RB200606‐04	S22°33′21.6″ W47°24′46.2″	Limeira‐SP	5.00E + 01	2.00E + 01	4.38E + 03	4.10E + 03	1.10E + 04
RB201102‐03	S22°21′56.5″ W46°56′53.1″	Mogi‐Guaçu‐SP	1.00E + 02	0.00E + 00	9.13E + 04	6.18E + 03	7.45Ev03
RB210802‐01	S22°43′33.0″ W47°39′38.5″	Piracicaba‐SP	—	—	—	—	7.00E + 05
RB210802‐02	S22°43′32.3″ W47°39′39.9″	Piracicaba‐SP	—	—	—	—	1.35E + 04
RB210802‐03	S22°43′32.3″ W47°39′39.8″	Piracicaba‐SP	—	—	—	—	3.50E + 03
RB210829‐01	S22°21′25.2″ W47°23′09.0″	Araras‐SP	—	—	—	—	2.15E + 03
RB210829‐02	S22°21′25.8″ W47°23′27.6″	Araras‐SP	—	—	—	—	2.45E + 03
RB210829‐03	S22°21′49.0″ W47°21′27.9″	Araras‐SP	—	—	—	—	1.55E + 02
RB210925‐01	S22°20′02.6″ W47°10′22.8″	Conchal‐SP	—	—	—	—	3.10E + 02
RB210925‐02	S22°22′37.7″ W46°56′26.0″	Mogi Guaçu‐SP	—	—	—	—	2.25E + 02
RB210925‐03	S22°24′08.2″ W46°56′59.4″	Mogi Mirim‐SP	—	—	—	—	2.50E + 01
RB210925‐04	S22°16′10.1″ W46°57′05.6″	Estiva Gerbi‐SP	—	—	—	—	2.75E + 03
RB210925‐05	S22°16′10.0″ W46°57′06.0″	Estiva Gerbi‐SP	—	—	—	—	3.95E + 03
RB211011‐01	S22°28′34.8″ W47°28′03.8″	Cordeirópolis‐SP	—	—	—	—	2.79E + 05
RB211011‐02	S22°28′34.2″ W47°28′03.5″	Cordeirópolis‐SP	—	—	—	—	2.21E + 05
RB211011‐04	S22°28′52.6″ W47°27′27.3″	Cordeirópolis‐SP	—	—	—	—	8.00E + 05
RB211011‐05	S22°28′51.6″ W47°27′26.4″	Cordeirópolis‐SP	—	—	—	—	3.85E + 04
RB211011‐06	S22°28′53.2″ W47°27′25.0″	Cordeirópolis‐SP	—	—	—	—	5.40E + 05
RB211017‐01	S23°32′52.1″ W46°38′35.6″	São Paulo‐SP	—	—	—	—	3.00E + 03
RB211017‐02	S23°33′43.5″ W46°39′26.2″	São Paulo‐SP	—	—	—	—	6.00E + 03
RB211017‐03	S23°35′45.5″ W46°42′44.4″	São Paulo‐SP	—	—	—	—	2.36E + 06
RB211017‐04	S23°35′40.1″ W46°42′56.1″	São Paulo‐SP	—	—	—	—	5.00E + 04
RB211017‐05	S23°35′39.7″ W46°42′56.2″	São Paulo‐SP	—	—	—	—	2.77E + 06

^a^ ME stands for external isolation and MI for internal isolation.

^b^ To compare the CFUs between infested or non‐infested fruits, we performed a Wilcoxon rank sum analysis with continuity correction. This was applied to the first set of yeast isolation (*n* = 10) of infested fruits, and the set recovered from non‐infested fruits that were artificially drilled (*n* = 2; 6.9E + 02 and 6.5E + 02).

We incubated plates at 20°C in darkness for 14 days and transferred yeast colonies to new YPD plates to obtain axenic cultures. We scored the number of colony‐forming units (CFUs). We stored cultures in 30% glycerol at −80°C and in solid GYMP (2% dextrose, 1% malt extract, 0.5% yeast extract, 0.2% NaH_2_PO_4_ and 1.5% agar) slants at 10°C as stocks. We deposited cultures in the UNESP Microbial Resource Center (CRM‐UNESP: WDCM 1043).

### 2.2 Yeast Barcoding

We extracted the genomic DNA of all yeast isolates following the methods described by Sampaio et al. (2001), with modifications in the incubation and centrifugation steps. Briefly, yeast cultures grown on YPD were suspended in 500 μL sterile lysis buffer (50 mM Tris, 50 mM EDTA, 250 mM NaCl and 0.3% SDS; pH 8) with glass beads (425–600 μm in diameter, Sigma‐Aldrich, St. Louis, MO, USA). Tubes were vortexed for 4 min and incubated at 65°C for 1 h (step repeated twice). Samples were centrifuged at 15,871 × g for 15 min, and 400 μL was transferred to sterile microtubes. We performed microsatellite‐primed polymerase chain reaction (MSP‐PCR) for each isolate, following Meyer et al. (1993), in 25 μL reactions. Reactions included 4 μL of 1.25 mM dNTPs (each), 5 μL of 5 × buffer, 2 μL of 25 mM MgCl_2_, 1.5 μL of 10 μM (GTG)_5_ primer, 7.3 μL ultrapure sterile water, 0.2 μL 5 U/μL^−1^ Taq polymerase, and 5 μL diluted DNA (1:750 μL in ultrapure sterile water). We conducted MSP‐PCR reactions as follows: 95°C for 3 min, 40 cycles at 93°C for 45 s, 50°C for 1 min, 72°C for 1 min, and a final extension step at 72°C for 6 min. We visualised the amplicons by electrophoresis for 3 h using 1.4% (w/v) agarose gel prepared with 0.5% TBE (Tris‐Borate‐EDTA) buffer. As a reference size marker, we used a 1 kb DNA ladder (Promega, Madison, WI, USA). We stained agarose gels with GelRed solution (16 μL 10,000 × GelRed, 0.56 g NaCl in 100 mL of ultrapure water) and visualised under UV. For each banding pattern, we selected at least two representative isolates for DNA sequencing, except for singletons.

For species delimitation, we amplified the D1/D2 region of the large subunit of the ribosomal (LSU) RNA gene using the primer pair NL1 and NL4 (Kurtzman and Robnett 1998) and the internal transcribed spacer (ITS) region using the primer pair ITS5 and ITS4 (White et al. 1990). Reactions included 4 μL of 1.25 mM dNTPs (each), 5 μL of 5 × buffer, 2 μL of 25 mM MgCl_2_, 1.0 μL of 10 μM primers, 6.8 μL ultrapure water, 0.2 μL 5 U μL^−1^ Taq polymerase, and 5 μL diluted DNA (1:750 μL in ultrapure water). We conducted PCR reactions as follows: LSU—96°C for 3 min, 35 cycles at 96°C for 30 s, 61°C for 45 s and 72°C for 1 min; ITS—96°C for 3 min, 35 cycles at 94°C for 1 min, 55°C for 1 min, and 72°C for 2 min.

We purified the amplicons with Wizard SV Gel and PCR Clean‐Up System (Promega, Madison, WI, USA) and sequenced using BigDye Terminator v. 3.1 Cycle Sequencing Kit (Thermo Fisher Scientific Inc., Waltham, MA, USA), following the manufacturer's protocols. We injected the amplicons into an ABI 3500 Series Genetic Analyser (Life Technologies, Carlsbad, CA, USA), and the generated forward and reversed sequences were assembled using BioEdit v.7.0.5.3 (Hall 1999). We deposited the contigs of the representative taxa in NCBI‐GenBank (https://www.ncbi.nlm.nih.gov/genbank/) under accessions PQ577697‐PQ577708 and PQ573566‐PQ573578 and compared with similar sequences deposited in the database by BLASTn (https://blast.ncbi.nlm.nih.gov/) with standard searching settings but limited for type strain sequences.

### 2.3 Yeast Phylogenetic Tree Reconstruction

For the phylogenetic reconstruction of yeast species, we aligned the datasets of ITS and LSU sequences with the closest relatives in MAFFT v.7.526 with the default automatic alignment method (Katoh et al. 2019). For final datasets, we concatenated ITS sequences with LSU sequence partitions. We calculated the nucleotide substitution models using ModelFinder (Kalyaanamoorthy et al. 2017) for each partition, using Bayesian information criterion with standard model generation in IQ‐TREE2 (details are listed in Table S1). We reconstructed maximum likelihood (ML) phylogenetic trees with ultrafast bootstrap approximation in IQTREE2 (Hoang et al. 2018; Minh et al. 2020; accession numbers of closest relatives used in phylogenetic analysis are listed in Table S2). Phylogenetic relationships were reconstructed with 10,000 replicates of ultrafast bootstrap (run parameters are detailed in Table S1). The final trees were edited in FigTree v.1.4.3 (Rambaut 2016).

### 2.4 Yeasts Associated With Beetle External Surfaces

To assess whether yeasts colonise the beetle's integument and fruit galleries, we examined male and female beetles and fruit samples under scanning electron microscopy (SEM). We fixed beetles and fragments of infested fruits (i.e., 2 cm^2^ squares) with the vapour of osmium tetroxide and then moved to aluminium stubs. We dehydrated the samples in acetone baths with increasing concentrations (i.e., 50%, 75%, 90%, 95% and 100%) until the critical point (Balzers CPD030). The material was stuck with double‐stick adhesive tape on stubs, coated with gold sputtering (Balzers SCD050) and examined in a scanning electron microscope (Hitachi TM3000). The presence of yeasts on the excavated front of males was specifically investigated, as it often accumulated fruit material.

### 2.5 Fruit Extract Assimilation by Recurrent Yeasts

To identify whether commonly associated yeasts are able to use the fruits as a solo carbon source or if they are inhibited by fruit compounds, we evaluated the ability of yeasts to survive and assimilate fruit extracts. We prepared extracts using 300 g of fruits. We crushed the fruits and incubated them for 2 days at 4°C in 1 L of water. Then, we filtered the extracts (pH = 3.5) with 0.22 μm cellulose filters and diluted them by ½, ¼ and ⅛. To make the assimilation media, we supplemented the dilutions with agar (15 g L^−1^), Yeast nitrogen base (YNB; BD Diagnostic Systems, Sparks, MD), and with or without the addition of dextrose (20 g L^−1^). As positive and negative controls, we prepared media without fruit extracts, and with or without dextrose (20 g L^−1^), respectively. A total of 48 representative yeast strains (listed in Table S3) were grown in fruit extracts as the only carbon source. We included *Saccharomyces cerevisiae* NCYC 1006 from the ale brewing process in the assay, as a control yeast not associated with this environment.

### 2.6 Yeast Volatile Choice Assay

To investigate whether yeast volatiles attract beetles, we suspended 10^8^ yeast cells mL^−1^ in 1× PBS and inoculated 30 mL of fruit extracts (prepared as previously described) in 125 mL Erlenmeyer flasks. We incubated the flasks overnight at 25°C under 150 rpm of rotation, followed by 2 days at 25°C under stationary conditions. We prepared an experimental setup with 9 cm diameter Petri dishes with traps equidistant from each other. We used the bulb of plastic Pasteur pipettes as traps (attaching them to the base of the Petri dishes at 0.5 cm distance from the edge, similar to pitfall traps). We laterally bored the Pasteur pipettes in both directions with 0.5 cm diameter holes allowing the beetles to enter but not leave the traps. We made a 0.5 cm diameter hole in the lids of the Petri dishes and used it for introducing the beetles to the experimental setup, which was kept without sealing to allow air circulation and volatile dissemination. We prepared fermented extracts as previously described and pipetted 300 μL into the traps (bulb of plastic Pasteur pipettes). Non‐fermented extracts and sterile water were used as positive and negative controls, respectively. We simultaneously placed females and males in the experimental setup, since both were collected together from the fruits, and kept them at room temperature for 12 h before the choice record (we regarded the absence of choice as one of the possibilities in the experimental setup).

We performed the first volatile choice assay with four traps, with two different yeasts and two controls. All yeast pairwise combinations between *Cyberlindnera* sp. B56, *Yamadazyma riverae* B343 and *Meyerozyma caribbica* B565 were evaluated (*Y. riverae* and *M. caribbica* were selected as they were the two most frequently occurring yeast species after *Cyberlindnera* sp.). We introduced a total of seven females and four males, all sampled from Rio Claro‐SP, to each experimental setup, and the assay was performed with five technical replicates for each yeast pairwise combination (a total of 35 females and 20 males for each trial). We performed a second volatile choice assay to compare the attraction of beetles towards *Cyberlindnera* sp. metabolites or its cells. Fruit extracts were prepared as previously described, and treatments were prepared by: removing yeast cells from extracts with 0.22 μm cellulose filters, cells resuspended in 1× PBS (recovering cells by centrifugation by 5000 × for 3 min—Eppendorf 5424—washing and resuspending in 1× PBS), keeping the cells together with the fermented extracts, and the two controls (non‐fermented extracts and sterile water, as previously described). We introduced a total of nine females and three males into the experimental setup with five traps with eight technical replicates (in a total of 72 females and 24 males). The difference in the number of males and females is due to the greater abundance of females on fruits. We recorded beetle choice as previously described.

### 2.7 Simulating Gallery Excavation by the Beetle

To confirm whether the yeasts were present in the fruits and not only transmitted by the beetles, we artificially produced galleries in the fruit with a sterile drill bit, simulating beetle excavation. We collected fruits in Rio Claro‐SP, superficially disinfected these with 70% ethanol, and with a disinfected drill bit (2 mm in diameter) a total of four holes were made in each fruit (5 mm in depth). We placed fruits in a plastic container with sand at room temperature and sprayed them with sterile water daily, simulating the natural conditions that fruits were exposed to by the beetles. We kept the fruits exposed to water or in dried conditions, with or without artificial holes. We used a total of eight fruits for each combination. We collected the fruits after 15 days, which were superficially disinfected with 70% ethanol and opened with sterile instruments. We removed the internal material of the fruits, combined it, and suspended it in 1 mL of 1× PBS. We diluted the samples ten‐fold and plated 100 μL in YPD supplemented with 150 μg mL^−1^ of chloramphenicol. We also inoculated the 100 μL suspensions on 125 mL Erlenmeyer flasks containing 25 mL of Yeast Extract–Peptone–Dextrose Broth (YPD without agar). We incubated the flasks at 25°C under 120 rpm of rotation over a week. We isolated and purified yeasts, and axenic cultures were identified as previously described (i.e., DNA extracted, followed by MSP‐PCR, and sequencing of D1/D2 barcoding region).

### 2.8 Statistical Analysis

To assess the differences in yeast numbers associated with male and female beetles, and fruits with or without beetles, we compared the CFUs using the Wilcoxon rank sum test with continuity correction and an alpha threshold of 0.05, since the data violated the parametric assumptions. We applied Bartlett and Shapiro–Wilk tests to check the homoscedasticity and normality of the data, respectively.

To assess the differences between beetle preference choices on ‘volatile choice assay’, we performed a Chi‐Square Goodness of Fit test comparing the recorded choices with the expected stochastic probability distribution. We performed the Exact binomial test with an alpha threshold of 0.05 as a post hoc test to compare the observed preferences with the expected stochastic distribution of each choice. Since the absence of choice was recorded as a possible choice in the experimental setup, we considered the stochastic nature to be 1/5 and 1/6, for the volatile choice assay performed with four and five traps, respectively. Additionally, we also exclude the absence of choice as a variable in the statistical analysis to validate consistency in the data analysis.

We conducted all analyses in RStudio v. 2023.12.0.369 (Posit team 2023) using R v.4.3.2 (R Core Team 2023) and ggplot2 package (Wickham 2016) for plot creation.

## 3 Results

### 3.1 Ironwood Fruits Infested by *Spermophthorus apuleiae* Harbour a Large Yeast Abundance

**FIGURE 1:**
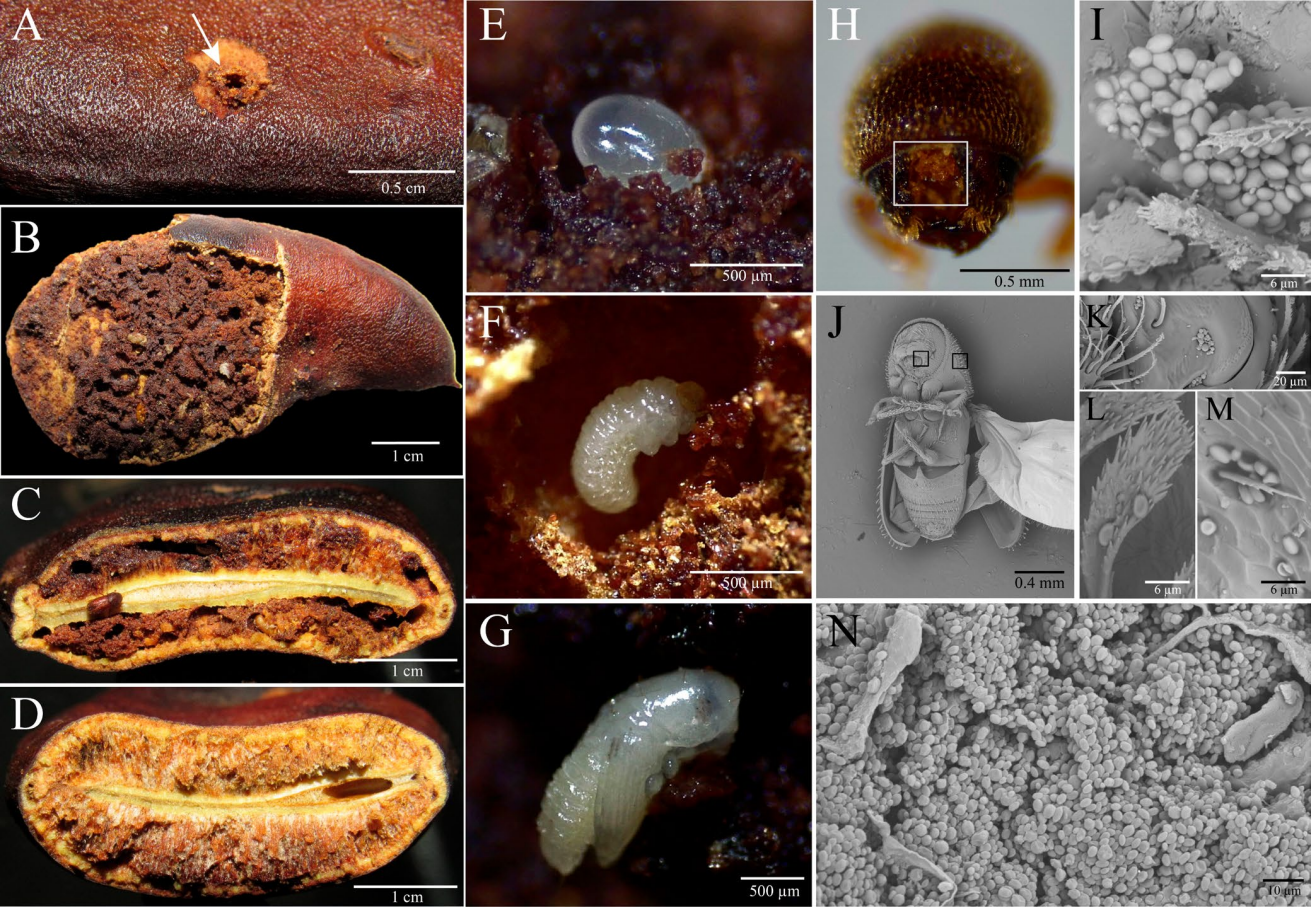
Interaction between *Spermophthorus apuleiae* and yeasts on *Libidibia ferrea* fruits. A. Gallery opening (arrow) in the fruit of *L. ferrea*. B. Opened fruit with structural alterations as a result of the beetle excavation. *C. side* view of fruit with structural alterations due to the beetle excavation. D. Fruit without the presence of beetles. E. Egg. F. Larva. and *G. pupa* found in the mesocarp of infested fruits. H. Frontal furrow of males (square) with accumulated fruit material. I. Yeasts found in males' frontal furrows by scanning electron microscopy (SEM). J. Beetle examined by scanning electron microscopy (SEM), squares indicated the locations where yeasts were found. K. Yeast cells found on the front part of the beetle. L. Yeast cells found attached to bristles by SEM. M. Yeast cells found in external elytra ornaments by SEM. N. Mesocarp of infested fruits with yeast cells in active galleries.

**FIGURE 2:**
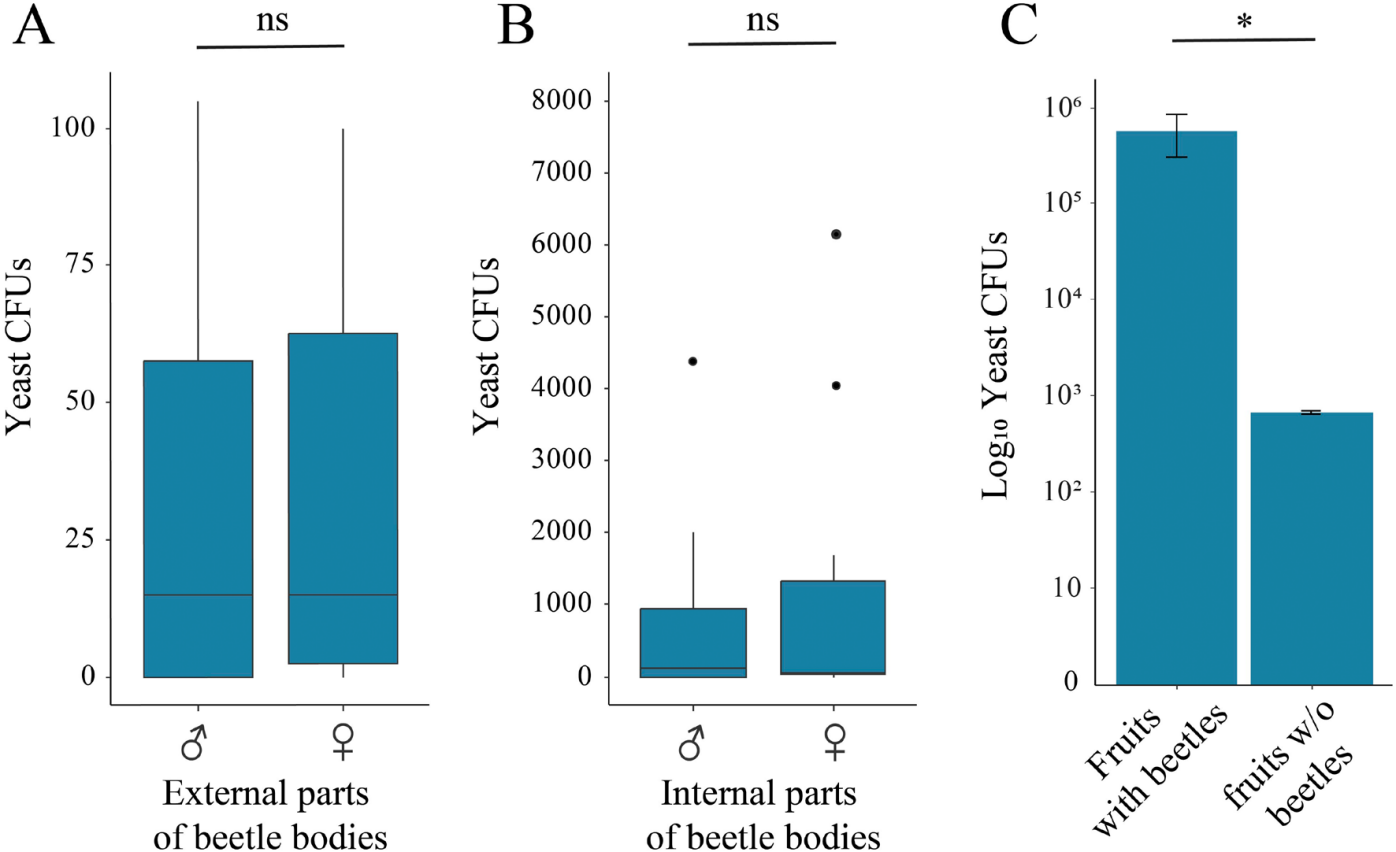
Abundance of yeasts by different isolation techniques. A. The number of yeasts found in external parts of beetle bodies in Colony Forming Units (CFUs) per mL. B. The number of yeasts in internal parts of beetle bodies in CFUs per mL. An outlier sample obtained internally from males (i.e., with 9.13 × 10^4^ yeast CFUs mL^−1^) was not represented for graphic reasons. C. Yeasts recovered from infested fruits (with beetles, *n* = 10) or by fruits submitted to artificial gallery constructions (drilling) under rainfall regimes simulation (w/o beetles, *n* = 2). Yeasts were not recovered from fruits without beetles and not submitted to artificial gallery constructions. Ns and asterisks respectively indicate not significantly and significantly different means for Wilcoxon rank sum test with continuity correction (*W* = 0, *p* = 0.04).

We observed that the mesocarp of the fruit was modified by beetles through gallery excavation for oviposition and larval development (Figure 1A–G), without damaging the seeds. Yeasts could be found in the external tegument of beetles, attached to bristles and ornaments of the exoskeleton (Figure 1H–M). Although the furrow on the frontal part of males accumulates fruit material (Figure 1H), the presence of yeasts was rarely found by SEM (Figure 1I), and our cultivation attempts of the accumulated fruit material resulted in no yeast growth. We confirmed the high density of yeasts in infested fruits with SEM (Figure 1N) and culture isolation using different sampling sources. The estimated number of yeast cells ranged from 10^1^ to 10^6^ CFUs mL^−1^ on the mesocarp of infested fruits, but from 0 to 10^2^ CFUs mL^−1^ on the external surfaces of the beetles and from 0 to 10^4^ CFUs mL^−1^ internally (Table 1, Figure 2A–C). Females and males do not differ in yeast counts for both internal and external isolation (Wilcoxon rank sum test with continuity correction, *W* = 49, *p* = 0.97, Figure 2A,B) but differ in yeast composition (Figure S1). We found no yeast in samples of non‐infested fruits. Moreover, in fruit extract assimilation assays, no yeast was inhibited by fruit extracts or had restriction in growth due to its use as sole carbon source in any of the evaluated concentrations.

### 3.2 *Cyberlindnera* sp. Was the Most Prevalent Yeast Associated With Fruits and Beetles

**FIGURE 3:**
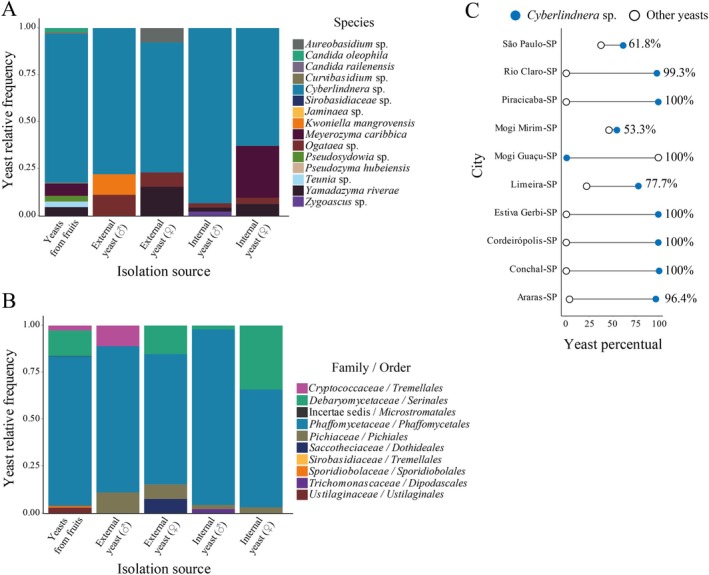
Taxonomy and occurrence of yeasts associated with *Spermophthorus apuleiae* and its environment. A. Relative frequency of yeast and yeast‐like species. B. Relative frequency of yeast and yeast‐like taxa sorted by family and order. C. Percentage of *Cyberlindnera* sp. compared to other yeast taxa in infested fruits sampled from different locations of São Paulo state.

We obtained 805 yeasts and yeast‐like isolates from 31 fruit samples and beetles, including 15 species distributed in 14 different genera (Figure 3A; Figures S2–S14; Table 2). We characterised yeast species by MSP‐PCR profiling, sequencing ribosomal RNA gene and spacers, and phylogenetic reconstruction. Most of the recovered yeasts and yeast‐like fungi belong to the phylum *Ascomycota*, with *Phaffomycetales* as the most frequent order, followed by *Serinales* (Figure 3B; with yeasts from *Saccharomycotina* subphylum). The yeast with affinities to the genus *Cyberlindnera* was the most recurrent species, representing 79% of the isolates (635 out of 805), and was found in 90% of the tree samples (28 out of 31). *Cyberlindnera* sp. was absent in three samples: two from Mogi‐Guaçu‐SP and one from São Paulo‐SP (Figure 3C). In these two samples from Mogi‐Guaçu‐SP, the most recurrent taxa in infested fruits were *Meyerozyma caribbica* (38 out of 59 isolates; 64%, a yeast from *Debaryomycetaceae* family), followed by *Pseudosydowia eucalypti* (18 out of 59 isolates; 31%, a yeast‐like from *Saccotheciaceae* family), while *Candida oleophila* was the most recurrent taxa in the São Paulo‐SP sample (4 out of 5 isolates). These figures were also reflected by the diversity indices which indicate a moderate diversity and uneven distribution of fungal species from fruit samples, highlighting the high frequency of *Cyberlindnera* sp. in this environment (Table S4).

**TABLE 2:** Yeast identification by barcoding.

Strain ID	Species	GenBank accession LSU/ITS	LSU‐blastn hit	ITS‐blastn hit	LSU/ITS identity %
B5	*Pseudozyma hubeiensis*	PQ573566/PQ577697	*Pseudozyma hubeiensis* (DQ008953)	*P. hubeiensis* (KY104687)	100.00/98.39
B127/LESF1535	*Ogataea* sp.	PP390556/PP390552	*Candida nanaspora* (KY106595)	*C. nanaspora* (NR_155574)	97.52/94.79
B343/LESF1538	*Yamadazyma riverae*	PQ573567/PQ577698	*Yamadazyma riverae* (NG_059986)	*Y. riverae* (NR_155968)	100.00/100.00
B437/LESF1872	*Aureobasidium* sp.	PQ573568/PQ577699	*Aureobasidium subglaciale* (MH874818)	*A. aerium* (NR_182588)	99.82/99.39
B455/LESF1848	*Curvibasidium* sp.	PQ573569/PQ577700	*Curvibasidium nothofagi* (NG_069005)	*C. nothofagi* (KY104889)	100.00/99.83
B477/LESF1510	*Zygoascus* sp.	PQ573570	*Zygoascus tannicola* (NG_058446)	—	94.92/−
B565/LESF1537	*Meyerozyma caribbica*	PQ573571/PQ577701	*Meyerozyma caribbica* (MH545919)	*M. caribbica* (MH545919)	100.00/100.00
B587/LESF1847	*Kwoniella mangrovensis*	PQ573572/PQ577702	*Kwoniella mangrovensis* (NG_042391)	*K. mangrovensis* (NR_073332)	100.00/98.84
B620/LESF1555	*Cyberlindnera* sp.	OQ253431/OQ268217	*Cyberlindnera rhodanensis* (NG_058753)	*C. samutprakarnensis*	88.79/81.80
B632/LESF1846	*Sirobasidiaceae* sp.	PQ573573/PQ577703	*Fibulobasidium inconspicuum* (NG_057677)	*F. murrhardtense* (NR_121466)	93.48/84.49
B633/LESF1873	*Jaminaea* sp.	PQ573574/PQ577704	*Jaminaea angkorensis* (NG_058308)	*J. angkorensis* (NR_155226)	99.49/97.59
B640/LESF1874	*Pseudosydowia* sp.	PQ573575/PQ577705	*Pseudosydowia louisecottisiae* (MW443079)	*P. phantasmae* (NR_171999)	98.95/91.20
B641/LESF1850	*Teunia* sp.	PQ573576/PQ577706	*Teunia globosa* (MK050288)	*T. virginiahalliae* (NR_191312)	98.33/92.43
B658/LESF1845	*Candida railenensis*	PQ573577/PQ577707	*Candida railenensis* (KY106716)	*C. railenensis* (NR_077080)	99.65/100.00
B660/LESF1849	*Candida oleophila*	PQ573578/PQ577708	*Candida oleophila* (NG_060820)	*C. oleophila* (NR_155224)	100.00/99.84

When non‐infested fruits were artificially drilled simulating gallery excavation by beetles, we recovered 6.70 × 10^2^ of *Cyberlindnera* sp. CFUs mL^−1^, but only when fruits were exposed to water (i.e., simulating rainfall regimes, which coincide in some months with *L. ferrea* fructification in São Paulo state, and predict to affect yeast counts mainly by precipitation and humidity, Figure 2C; Figure S15). We recover an average of 5.74 × 10^5^ of yeast CFUs mL^−1^ on naturally infested fruits, an 800‐fold CFUs increase compared with artificially drilled fruits (Figure 2C, Table 1; Wilcoxon rank sum test with continuity correction, *W* = 0, *p* = 0.04). No yeast isolates were recovered in treatments where drilled or not drilled fruits were kept without exposure to water.

### 3.3 *Cyberlindnera* sp. Volatiles Attract Beetles

**FIGURE 4:**
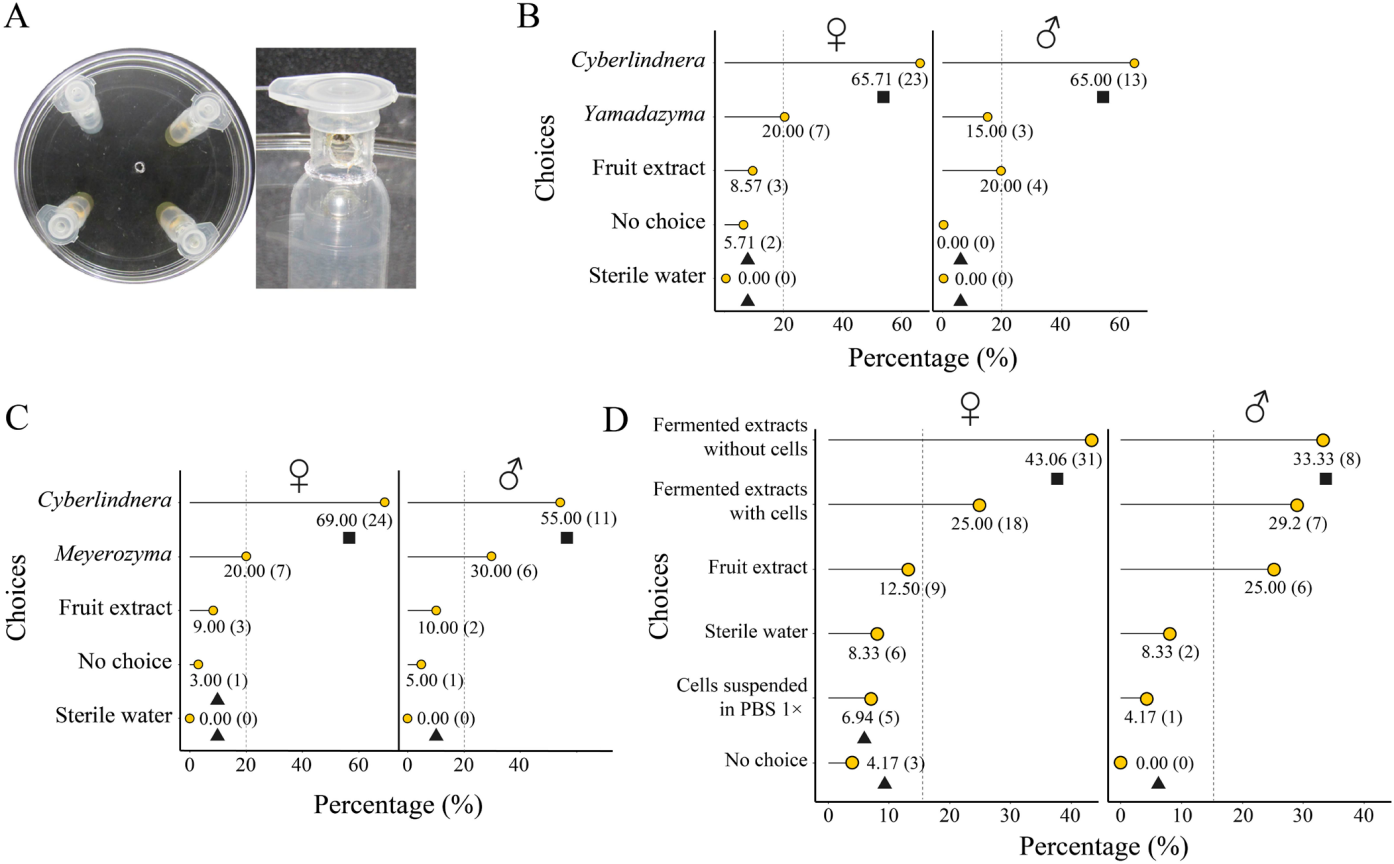
Preference of beetles towards *Cyberlindnera* volatiles. A. Experimental set‐up used to assess beetle preference. Percentage of preference of beetles to volatile metabolites of *Cyberlindnera* sp. B56 in relation to B. *Yamadazyma riverae* B343 and C. *Meyerozyma caribbica* B565. D. Percentage of preference of beetles to volatile metabolites from fermented fruit extracts containing or not *Cyberlindnera* sp. B56 cells. Absolute numbers are shown in parentheses. Black squares and triangles represent values higher and lower than expected by a stochastic distribution, respectively (details in Table S7). Dotted lines indicate stochastic probabilities for the choice of beetles in each experiment. Abbreviations on the y‐axis: ‘*Cyberlindnera*’ – *Cyberlindnera* sp. B56 (fermented extract), ‘*Meyerozyma*’ – *Meyerozyma caribbica* B565 (fermented extract), ‘*Yamadazyma*’ – *Yamadazyma riverae* B343 (fermented extract), ‘Cells suspended in PBS 1×’ – *Cyberlindnera* sp. cells recovered and suspended in PBS 1×, ‘Fermented extracts with cells’ – Fermented fruit extract containing *Cyberlindnera* sp. B56 cells, and ‘Fermented extracts without cells’ – Fruit extract fermented by *Cyberlindnera* sp. B56 and filtered (removing cells by 0.22 μm culture filtration).

Using our experimental set‐up, we observed that beetles were attracted towards yeast volatiles (Figure 4A–D), with a preference for *Cyberlindnera* sp. volatiles. Fruit extracts fermented by *Cyberlindnera* sp., containing the yeast cells or not, were the most attractive choice for both females and males (Figure 4D), attracting 68.06% and 62.50% of the beetles, respectively (Tables S5–S7; *Exact binomial test*, *p* < 0.05). Assays comparing the different yeasts indicate the beetles' preference for *Cyberlindnera* sp. volatiles over *Yamadazyma riverae* and *Meyerozyma caribbica*, which attracted at least 55% of females and males in all combinations (Tables S5–S7; Figure 4B,C, *Exact binomial test*, *p* < 0.05). When *Cyberlindnera* sp. was not provided as a choice, treatments that do not contain yeasts (i.e., fruit extract, water, or no choice) increased in choice, reaching 45.71% of females and 45.00% of males (Tables S5–S7). Only males showed attraction to volatiles of *M. caribbica* over *Y. riverae*, when in the absence of *Cyberlindnera* sp. as a choice (Tables S5–S7; *Exact binomial test*, *p* < 0,05). When the absence of choice was not recorded as a variable, minor differences were observed, including: no significant differences for males in the second volatile choice assay (*Chi‐Square Goodness of Fit test*, X‐squared = 8.083; df = 4; *p* = 0.089; Table S8) and a lack of attraction of males to *M. caribbica* volatiles in the absence of *Cyberlindnera* sp. (*Exact binomial test*, *p* > 0.05; Table S9).

## 4 Discussion

Using culture‐dependent methods, we provide evidence for a previously unrecognised interaction between the borer beetle *S. apuleiae*, the fruits of *L. ferrea*, and yeasts with affinity for the genus *Cyberlindnera*. Our findings show that beetles not only have a frugivorous lifestyle as previously thought (Suesdek and Lima 2011) but also ingest yeast cells present in the galleries built in the fruits. Although the excavated frons of males often accumulate fruit material, yeasts were rarely detected in this substrate, suggesting that this morphological trait is not directly associated with yeast transport, but it may be related to other functional traits common to *Spermophthorus* species and other Pityophthorina species (S. L. Wood 1982, 2007). Sexual dimorphism is especially common in the frons of bark and ambrosia beetles and is presumed to be indirectly involved in mating and species recognition (Kirkendall et al. 2015).

Beetle tunnelling behaviour alters the fruit environment, enhancing the growth of *Cyberlindnera* sp., which is naturally present in non‐infested fruits but at low density. We also demonstrate that *Cyberlindnera* sp. produces volatiles that are attractive and distinctive to the beetle, using fruit extracts as substrate. Yeast cells were recovered from the exterior and interior of the beetle bodies, further supporting a possible yeast vectoring. Together, our findings indicate an interaction between two organisms with positive feedback, under certain conditions and during a particular stage of their life cycles, with mutual modifications in their shared niche. Thus, we hypothesise that this is a case of a facultative mutualism. Since both partners were found in the absence of each other (i.e., the yeast is autochthonous in fruits, and in few cases, beetles were found without *Cyberlindnera*), we also define this interaction so far as a case of non‐symbiotic interaction, which still requires further elucidation. Future studies assessing partner fitness may provide elements to determine the reciprocal benefits of partners and traits subjected to strong natural selection.

*Spermophthorus apuleiae* is known to build galleries in *L. ferrea* fruits (A. Costa Lima 1929; A. M. Costa Lima 1956) without causing harm to the seeds (Suesdek and Lima 2011); however, this interaction remains poorly understood. Although *L. ferrea* is a widespread native tree in Brazil (Oliveira and Fernando 2024), mainly occurring in the northeast of the country (Carvalho 2010; Oliveira and Fernando 2024) and extensively used in urban afforestation (Almeida et al. 2021; de Carvalho Maria et al. 2020), it remains unclear whether geography or environmental conditions influence this interaction. All samples analysed in this study were collected from trees located in urban environments; therefore, the dynamics of this interaction in natural settings still require further investigation. Likewise, it remains to be determined whether the recently recognised *L. ferrea* species complex (Oliveira et al. 2024) influences the presence of *Cyberlindnera* sp., potentially explaining its absence in some cases.

The yeast with affinities to the genus *Cyberlindnera* has so far only been found in this environment. However, it belongs to the family *Phaffomycetaceae*—which includes the genera *Barnettozyma*, *Cyberlindnera*, *Phaffomyces* and *Starmera*—a group with several members known to associate with insects or insects' habitats (Kurtzman 2011a, 2011b; Mestre et al. 2011; Urbina et al. 2013). Within this family, insect‐yeast associations have been documented in various beetle species. *Phaffomyces* species have been isolated from the gut of the passalid beetle *Ogyges laevissimus* (Urbina et al. 2013). *Cyberlindnera americana* has been found in association with multiple developmental stages of *Dendroctonus* beetles (Dohet et al. 2016; Briones‐Roblero et al. 2017). In addition, *Cyberlindnera* spp. have been recovered from mycetangia of *Elateroides* spp. beetles (Toki 2021, 2023).

Yeasts contribute to insect survival either directly, by providing nutritional benefits from yeast cells, or indirectly by yeast metabolism, detoxifying harmful plant allelochemicals in their environment (Starmer and Fogleman 1986; Dowd and Shen 1990). A particular example of this detoxification role involves the beetles *Stegobium paniceum* and *Lasioderma serricorne* , which associate with yeast‐like symbionts of the genus *Symbiotaphrina* (Martinson 2020). In particular, *S. kochii*, a symbiont of *L. serricorne* , degrades several plant allelochemicals, toxins, insecticides and herbicides (including the plant phenolics, such as gallic acid; Shen and Dowd 1989), by producing detoxification enzymes and utilising these compounds as carbon sources (Shen and Dowd 1991). Thus, the yeast‐like partner contributes to host survival, since symbiont‐free beetles face high mortality and delayed development when fed on diets containing plant allelochemicals (Dowd and Shen 1990).

Here, we found that despite the concentration of fruit extracts, yeasts were observed to grow and are not inhibited on media using the extracts as a sole carbon source. Ellagic and gallic acids are the most recurrent phenolic compounds found in *L. ferrea* fruits (Ueda et al. 2001; Nakamura et al. 2002; Ferreira et al. 2016; Comandolli‐Wyrepkowski et al. 2017), and together with tannins play a defensive role against herbivores (Ananthakrishnan 1997). We hypothesise that yeasts may contribute to chemical detoxification, rendering the fruit more palatable to the beetle. Over time, this could drive the beetle to adapt to the yeast‐modified environment, a pattern also observed in yeast‐*Drosophila* interactions (Buser et al. 2014). Alternatively, beetle gut enzymes or other microbial symbionts may play a role in environmental detoxification (Adams et al. 2013; Ashraf et al. 2023). Future trials should consider yeast‐free beetles fed with yeast‐digested or non‐digested fruit extracts, as well as screening the yeast enzymatic profile, and the use of yeasts as nourishment sources by the larvae.

The insect gut of several insect orders is reported as a suitable environment for yeast survival (Stefanini 2018). Faecal materials can contain yeast viable cells, which contribute to larval development, as in the fruit fly *Drosophila melanogaster* (Cho and Rohlfs 2023). In some beetle species from Curculionidae, Languriinae, Lucanidae and Lymexylidae families, yeast cells can be hosted in mycetangia (Davis et al. 2011; Toki et al. 2012; Toki 2021, 2023; Yamamoto and Toki 2023). Here, even though we isolated yeasts from the internal parts of the beetle body, the efforts were not targeted at a specific region, such as the gut or any other internal organs that resemble mycetangia. We consider that future efforts involving the search for yeasts in beetle guts and faeces, as well as for structures in the beetle's bodies that can host yeast cells, will provide clues if *Cyberlindnera* sp. can be maintained in this system by the beetles. Recently, we explored the rearrangements of rDNA repeat arrays in this yeast with affinities to *Cyberlindnera* (Bizarria et al. 2023), which can have its sexual state induced in culture. Given that, interspecies hybridisation could lead to the unilateral acquisition (like in *Pichia sorbitophila*, Louis et al. 2012) or the inheritance of both parents' rDNA repeat units (like in *Zygosaccharomyces rouxii*, Gordon and Wolfe 2008). Since mating and hybridisation are believed to be benefits of yeasts associated with insects (Reuter et al. 2007; Stefanini et al. 2016), we hypothesise that the sexual reproduction of *Cyberlindnera* sp. can have a role in the rDNA arrays polymorphisms (Bizarria et al. 2023), which should be further investigated in the light of this insect‐yeast interaction.

Insect attraction to yeast volatiles has been demonstrated in different insect species, including beetles (Torto et al. 2007; Benda et al. 2008; Baig et al. 2020). This attraction has been hypothesized to have evolved as chemical signals of sugar resources, enhancing yeast dispersal among sugary environments by the insects (‘dispersal–encounter hypothesis’, Madden et al. 2018), and/or contribute to the evolution of angiosperm pollination mediated by insects (Becher et al. 2018). We demonstrate that *S. apuleiae* beetles are attracted by *Cyberlindnera* sp. volatiles, which may be an important clue for the specificity of some yeast‐insect interactions (Madden et al. 2018). The volatile signals released by yeasts are also known to affect insect behaviours (e.g., oviposition in the fruit fly *Drosophila melanogaster* , Becher et al. 2012; anti‐aggregation in the mountain pine beetle *Dendroctonus ponderosae* , Hunt and Borden 1990; and oviposition in the codling moth *Cydia pomonella*, Witzgall et al. 2012, are some of the examples), and to affect the growth of other associated microbes (Adams et al. 2008; Davis et al. 2011; Chakraborty et al. 2022). Another possibility is that the volatiles are by‐product metabolites acting in other biological processes involved in the niche construction (Buser et al. 2014 and references within). Future efforts should elucidate the chemical profile of those volatile emissions by the yeast and its exclusive attractiveness to this beetle or even other insects.

Overall, we demonstrated that *S. apuleiae* beetles modify the structure of *L. ferrea* fruit mesocarp, increasing the population growth of *Cyberlindnera* sp., and that the yeast produces volatile compounds that are attractive to the beetles. Considering that the *L. ferrea* is widespread in Brazil, having trackable fruiting regimes in specific seasons, and with the clues for the interaction between *Cyberlindnera* sp. and the borer beetle *S. apuleiae* in *L. ferrea* fruits, we consider that this system could be a prominent model for studying yeast‐insect interactions, with an avenue of uncertainties and challenges.

## Author Contributions

**Rodolfo Bizarria Jr.:** conceptualization, investigation, writing – original draft, methodology, validation, writing – review and editing, formal analysis, data curation. **Tatiane de Castro Pietrobon:** writing – review and editing, methodology, data curation. **Pepijn W. Kooij:** writing – review and editing, methodology. **Andre Rodrigues:** conceptualization, writing – original draft, funding acquisition, writing – review and editing, methodology, supervision.

## Conflicts of Interest

The authors declare no conflicts of interest.

## Supporting information


Data S1.

## Data Availability

Sequence data is available from GenBank under accessions PQ577697–PQ577708; PQ573566–PQ573578. The data supporting the results in the paper are provided as electronic Supporting Information.

## References

[emi470157-bib-0002] Adams, A. S. , D. L. Six , S. M. Adams , and W. E. Holben . 2008. “In Vitro Interactions Between Yeasts and Bacteria and the Fungal Symbionts of the Mountain Pine Beetle ( *Dendroctonus ponderosae* ).” Microbial Ecology 56: 460–466. 10.1007/s00248-008-9364-0. 18322728

[emi470157-bib-0001] Adams, A. S. , F. O. Aylward , S. M. Adams , et al. 2013. “Mountain Pine Beetles Colonizing Historical and Naive Host Trees Are Associated With a Bacterial Community Highly Enriched in Genes Contributing to Terpene Metabolism.” Applied and Environmental Microbiology 79, no. 11: 3468–3475. 10.1128/AEM.00068-13. 23542624 PMC3648045

[emi470157-bib-0003] Almeida, N. C. O. d. S. , S. d. C. Furtado , and J. F. M. Barcellos . 2021. “A Narrative Review of *Libidibia ferrea*: Botanical Aspects, Ethnopharmacological Properties, Phytochemical Characteristics, Toxicity, and Experimental Tests.” European Journal of Medicinal Plants 32, no. 12: 16–30. 10.9734/ejmp/2021/v32i1230432.

[emi470157-bib-0004] Ananthakrishnan, T. N. 1997. “Gallic and Salicylic Acids: Sentinels of Plant Defence Against Insects.” Current Science 73, no. 7: 576–579.

[emi470157-bib-0088] Arcuri, S. L. , F. C. Pagnocca , W. G. da Paixão Melo , N. S. Nagamoto , D. L. Komura , and A. Rodrigues . 2014. “Yeasts Found on an Ephemeral Reproductive Caste of the Leaf‐cutting Ant *Atta sexdens rubropilosa* .” Antonie van Leeuwenhoek 106, no. 3: 475–487. 10.1007/s10482-014-0216-2. 25012689

[emi470157-bib-0005] Ashraf, M. Z. , K. Mogilicherla , G. Sellamuthu , V. Siino , F. Levander , and A. Roy . 2023. “Comparative Gut Proteomics Study Revealing Adaptive Physiology of Eurasian Spruce Bark Beetle, *Ips typographus* (Coleoptera: Scolytinae).” Frontiers in Plant Science 14: 1157455. 10.3389/fpls.2023.1157455. 38078109 PMC10703158

[emi470157-bib-0006] Asplund, J. , and D. A. Wardle . 2017. “How Lichens Impact on Terrestrial Community and Ecosystem Properties.” Biological Reviews 92, no. 3: 1720–1738. 10.1111/brv.12305. 27730713

[emi470157-bib-0007] Baig, F. , K. Farnier , A. M. Piper , R. Speight , and J. P. Cunningham . 2020. “Yeasts Influence Host Selection and Larval Fitness in Two Frugivorous *Carpophilus* Beetle Species.” Journal of Chemical Ecology 46: 675–687. 10.1007/s10886-020-01167-5. 32185581

[emi470157-bib-0009] Becher, P. G. , A. Hagman , V. Verschut , et al. 2018. “Chemical Signaling and Insect Attraction Is a Conserved Trait in Yeasts.” Ecology and Evolution 8, no. 5: 2962–2974. 10.1002/ece3.3905. 29531709 PMC5838033

[emi470157-bib-0008] Becher, P. G. , G. Flick , E. Rozpędowska , et al. 2012. “Yeast, Not Fruit Volatiles Mediate *Drosophila melanogaster* Attraction, Oviposition and Development.” Functional Ecology 26, no. 4: 822–828. 10.1111/j.1365-2435.2012.02006.x.

[emi470157-bib-0010] Bellutti, N. , A. Gallmetzer , G. Innerebner , S. Schmidt , R. Zelger , and E. H. Koschier . 2018. “Dietary Yeast Affects Preference and Performance in *Drosophila suzukii* .” Journal of Pest Science 91: 651–660. 10.1007/s10340-017-0932-2. 29568250 PMC5847167

[emi470157-bib-0011] Benda, N. D. , D. Boucias , B. Torto , and P. Teal . 2008. “Detection and Characterization of *Kodamaea ohmeri* Associated With Small Hive Beetle *Aethina tumida* Infesting Honey Bee Hives.” Journal of Apicultural Research 47, no. 3: 194–201. 10.3827/IBRA.1.47.3.07.

[emi470157-bib-0012] Bizarria, R. , T. de Castro Pietrobon , H. Ferreira , and A. Rodrigues . 2023. “Polymorphisms of rDNA Genes in *Cyberlindnera* Yeast Suggest Birth and Death Evolution Events.” FEMS Yeast Research 23: foad032. 10.1093/femsyr/foad032. 37291697

[emi470157-bib-0013] Boucher, D. H. , S. James , and K. H. Keeler . 1982. “The Ecology of Mutualism.” Annual Review of Ecology and Systematics 13, no. 1: 315–347.

[emi470157-bib-0014] Briones‐Roblero, C. I. , R. Rodríguez‐Díaz , J. A. Santiago‐Cruz , G. Zúñiga , and F. N. Rivera‐Orduña . 2017. “Degradation Capacities of Bacteria and Yeasts Isolated From the Gut of *Dendroctonus rhizophagus* (Curculionidae: Scolytinae).” Folia Microbiologica 62: 1–9. 10.1007/s12223-016-0469-4. 27544667

[emi470157-bib-0015] Buser, C. C. , R. D. Newcomb , A. C. Gaskett , and M. R. Goddard . 2014. “Niche Construction Initiates the Evolution of Mutualistic Interactions.” Ecology Letters 17, no. 10: 1257–1264. 10.1111/ele.12331. 25041133

[emi470157-bib-0016] Carvalho, P. E. R. 2010. Espécies Arbóreas Brasileiras. Vol. 4. EMBRAPA Informação Tecnológica.

[emi470157-bib-0017] Chakraborty, A. , B. Mori , G. Rehermann , et al. 2022. “Yeast and Fruit Fly Mutual Niche Construction and Antagonism Against Mould.” Functional Ecology 36, no. 7: 1639–1654. 10.1111/1365-2435.14054.

[emi470157-bib-0018] Cho, H. , and M. Rohlfs . 2023. “Transmission of Beneficial Yeasts Accompanies Offspring Production in *Drosophila*—An Initial Evolutionary Stage of Insect Maternal Care Through Manipulation of Microbial Load?” Ecology and Evolution 13, no. 6: e10184. 10.1002/ece3.10184. 37332518 PMC10276349

[emi470157-bib-0019] Chomicki, G. , M. Weber , B. B. Grafova , J. Bascompte , and E. T. Kiers . 2019. “The Impact of Mutualisms on Species Richness.” Trends in Ecology & Evolution 34, no. 8: 698–711. 10.1016/j.tree.2019.03.003. 31003875

[emi470157-bib-0020] Comandolli‐Wyrepkowski, C. D. , C. B. Jensen , G. Rehermann , et al. 2017. “Antileishmanial Activity of Extracts From *Libidibia ferrea*: Development of In Vitro and In Vivo Tests.” Acta Amazonica 47: 331–340. 10.1590/1809-4392201700871.

[emi470157-bib-0021] Costa Lima, A. 1929. “Sobre Dois Scolytideos.” Memórias do Instituto Oswaldo Cruz 22: 109–112.

[emi470157-bib-0022] Costa Lima, A. M. 1956. Insetos do Brasil: Coleópteros. Vol. 10. Escola Nacional de Agronomia.

[emi470157-bib-0023] Davis, T. S. , R. W. Hofstetter , J. T. Foster , N. E. Foote , and P. Keim . 2011. “Interactions Between the Yeast *Ogataea pini* and Filamentous Fungi Associated With the Western Pine Beetle.” Microbial Ecology 61: 626–634. 10.1007/s00248-010-9773-8. 21085946

[emi470157-bib-0024] de Carvalho Maria, T. R. B. , B. F. H. Bomm , J. Nesi , T. L. Ho , and R. Bobrowski . 2020. “Canopy Architecture and Morphometry of Tree Species Used in the Urban Forest.” Floresta 50, no. 4: 1892–1901.

[emi470157-bib-0025] Dohet, L. , J. C. Gregoire , A. Berasategui , M. Kaltenpoth , and P. H. Biedermann . 2016. “Bacterial and Fungal Symbionts of Parasitic *Dendroctonus* Bark Beetles.” FEMS Microbiology Ecology 92, no. 9: fiw129. 10.1093/femsec/fiw129. 27387908

[emi470157-bib-0026] Dowd, P. F. , and S. K. Shen . 1990. “The Contribution of Symbiotic Yeast to Toxin Resistance of the Cigarette Beetle ( *Lasioderma serricorne* ).” Entomologia Experimentalis et Applicata 56, no. 3: 241–248. 10.1111/j.1570-7458.1990.tb01402.x.

[emi470157-bib-0027] Ferreira, M. R. , M. T. Fernandes , W. A. da Silva , et al. 2016. “Chromatographic and Spectrophotometric Analysis of Phenolic Compounds From Fruits of *Libidibia ferrea* Martius.” Pharmacognosy Magazine 12, no. Suppl 2: S285. 10.4103/0973-1296.182165. 27279721 PMC4883093

[emi470157-bib-0028] Ganter, P. F. 1988. “The Vectoring of Cactophilic Yeasts by *Drosophila* .” Oecologia 75, no. 3: 400–404. 10.1007/BF00376943. 28312688

[emi470157-bib-0029] Gilbert, D. G. 1980. “Dispersal of Yeasts and Bacteria by *Drosophila* in a Temperate Forest.” Oecologia 46: 135–137. 10.1007/BF00346979. 28310639

[emi470157-bib-0030] Gordon, J. L. , and K. H. Wolfe . 2008. “Recent Allopolyploid Origin of *Zygosaccharomyces rouxii* Strain ATCC 42981.” Yeast 25, no. 6: 449–456. 10.1002/yea.1598. 18509846

[emi470157-bib-0031] Hall, T. A. 1999. “BioEdit: A User‐Friendly Biological Sequence Alignment Editor and Analysis Program for Windows 95/98/NT.” Nucleic Acids Symposium Series 41: 95–98.

[emi470157-bib-0032] Hoang, D. T. , O. Chernomor , A. Von Haeseler , B. Q. Minh , and L. S. Vinh . 2018. “UFBoot2: Improving the Ultrafast Bootstrap Approximation.” Molecular Biology and Evolution 35, no. 2: 518–522. 10.1093/molbev/msx281. 29077904 PMC5850222

[emi470157-bib-0033] Howe, H. F. 1984. “Constraints on the Evolution of Mutualisms.” American Naturalist 123, no. 6: 764–777. 10.1086/284238.

[emi470157-bib-0034] Hunt, D. W. A. , and J. H. Borden . 1990. “Conversion of Verbenols to Verbenone by Yeasts Isolated From *Dendroctonus ponderosae* (Coleoptera: Scolytidae).” Journal of Chemical Ecology 16, no. 4: 1385–1397. 10.1007/BF01021034. 24263735

[emi470157-bib-0035] Kalyaanamoorthy, S. , B. Q. Minh , T. K. Wong , A. Von Haeseler , and L. S. Jermiin . 2017. “ModelFinder: Fast Model Selection for Accurate Phylogenetic Estimates.” Nature Methods 14, no. 6: 587–589. 10.1038/nmeth.4285. 28481363 PMC5453245

[emi470157-bib-0036] Katoh, K. , J. Rozewicki , and K. D. Yamada . 2019. “MAFFT Online Service: Multiple Sequence Alignment, Interactive Sequence Choice and Visualization.” Briefings in Bioinformatics 20, no. 4: 1160–1166. 10.1093/bib/bbx108. 28968734 PMC6781576

[emi470157-bib-0037] Keeler, K. H. 1981. “A Model of Selection for Facultative Nonsymbiotic Mutualism.” American Naturalist 118, no. 4: 488–498. 10.1086/283843.

[emi470157-bib-0038] Kirkendall, L. R. , P. H. Biedermann , and B. H. Jordal . 2015. “Evolution and Diversity of Bark and Ambrosia Beetles.” In Bark Beetles, edited by F. E. Vega and R. W. Hofstetter , 85–156. Academic Press. 10.1016/B978-0-12-417156-5.00003-4.

[emi470157-bib-0039] Koerte, S. , I. W. Keesey , M. L. Easson , J. Gershenzon , B. S. Hansson , and M. Knaden . 2020. “Variable Dependency on Associated Yeast Communities Influences Host Range in *Drosophila Species* .” Oikos 129, no. 7: 964–982. 10.1111/oik.07180.

[emi470157-bib-0040] Kurtzman, C. P. 2011a. “ *Barnettozyma* Kurtzman, Robnett, Basehoar‐Powers (2008).” In The Yeasts, 333–339. Elsevier.

[emi470157-bib-0041] Kurtzman, C. P. 2011b. “ *Lindnera* Kurtzman, Robnett, Basehoar‐Powers (2008).” In The Yeasts, 521–543. Elsevier.

[emi470157-bib-0042] Kurtzman, C. P. , and C. J. Robnett . 1998. “Identification and Phylogeny of Ascomycetous Yeasts From Analysis of Nuclear Large Subunit (26S) Ribosomal DNA Partial Sequences.” Antonie Van Leeuwenhoek 73, no. 4: 331–371. 10.1023/A:1001761008817. 9850420

[emi470157-bib-0043] Lachance, M. A. , W. T. Starmer , C. A. Rosa , J. M. Bowles , J. S. F. Barker , and D. H. Janzen . 2001. “Biogeography of the Yeasts of Ephemeral Flowers and Their Insects.” FEMS Yeast Research 1, no. 1: 1–8. 10.1016/S1567-1356(00)00003-9. 12702457

[emi470157-bib-0044] Lou, Q. Z. , M. Lu , and J. H. Sun . 2014. “Yeast Diversity Associated With Invasive *Dendroctonus valens* Killing *Pinus tabuliformis* in China Using Culturing and Molecular Methods.” Microbial Ecology 68: 397–415. 10.1007/s00248-014-0413-6. 24691849

[emi470157-bib-0045] Louis, V. L. , L. Despons , A. Friedrich , et al. 2012. “ *Pichia sorbitophila*, an Interspecies Yeast Hybrid, Reveals Early Steps of Genome Resolution After Polyploidization.” G3: Genes, Genomes, Genetics 2, no. 2: 299–311. 10.1534/g3.111.000745. 22384408 PMC3284337

[emi470157-bib-0046] Madden, A. A. , M. J. Epps , T. Fukami , et al. 2018. “The Ecology of Insect–Yeast Relationships and Its Relevance to Human Industry.” Proceedings of the Royal Society B: Biological Sciences 285, no. 1875: 20172733. 10.1098/rspb.2017.2733. PMC5897634 29563264

[emi470157-bib-0047] Martinson, V. G. 2020. “Rediscovering a Forgotten System of Symbiosis: Historical Perspective and Future Potential.” Genes 11, no. 9: 1063. 10.3390/genes11091063. 32916942 PMC7563122

[emi470157-bib-0048] Mestre, M. C. , C. A. Rosa , and S. B. Fontenla . 2011. “ *Lindnera rhizosphaerae* sp. Nov., a Yeast Species Isolated From Rhizospheric Soil.” International Journal of Systematic and Evolutionary Microbiology 61, no. 4: 985–988. 10.1099/ijs.0.022863-0. 20418411

[emi470157-bib-0049] Meyer, S. T. , I. R. Leal , M. Tabarelli , and R. Wirth . 2011. “Ecosystem Engineering by Leaf‐Cutting Ants: Nests of *Atta cephalotes* Drastically Alter Forest Structure and Microclimate.” Ecological Entomology 36, no. 1: 14–24. 10.1111/j.1365-2311.2010.01241.x.

[emi470157-bib-0050] Meyer, W. , T. G. Mitchell , E. Z. Freedman , and R. Vilgalys . 1993. “Hybridization Probes for Conventional DNA Fingerprinting Used as Single Primers in the Polymerase Chain Reaction to Distinguish Strains of *Cryptococcus neoformans* .” Journal of Clinical Microbiology 31, no. 9: 2274–2280. 10.1128/jcm.31.9.2274-2280.1993. 8408543 PMC265746

[emi470157-bib-0051] Minh, B. Q. , H. A. Schmidt , O. Chernomor , et al. 2020. “IQ‐TREE 2: New Models and Efficient Methods for Phylogenetic Inference in the Genomic Era.” Molecular Biology and Evolution 37, no. 5: 1530–1534. 10.1093/molbev/msaa015. 32011700 PMC7182206

[emi470157-bib-0052] Nakamura, E. S. , F. Kurosaki , M. Arisawa , et al. 2002. “Cancer Chemopreventive Effects of a Brasilian Folk Medicine, Juca, on In Vivo Two‐Stage Skin Carcinogenesis.” Journal of Ethnopharmacology 81, no. 1: 135–137. 10.1016/s0378-8741(02)00047-8. 12020938

[emi470157-bib-0053] Nguyen, N. H. , S. O. Suh , C. K. Erbil , and M. Blackwell . 2006. “ *Metschnikowia noctiluminum* sp. Nov., *Metschnikowia corniflorae* sp. Nov., and *Candida chrysomelidarum* sp. Nov., Isolated From Green Lacewings and Beetles.” Mycological Research 110, no. 3: 346–356. 10.1016/j.mycres.2005.11.010. 16483756

[emi470157-bib-0054] Oliveira, F. G. , and E. M. P. Fernando . 2024. Libidibia in Flora e Funga do Brasil. Jardim Botânico do Rio de Janeiro. https://floradobrasil.jbrj.gov.br/FB109828.

[emi470157-bib-0055] Oliveira, F. G. , F. D. S. Santos , G. P. Lewis , R. P. Oliveira , and L. P. Queiroz . 2024. “Reassessing the Taxonomy of the *Libidibia ferrea* Complex, the Iconic Brazilian Tree “Pau‐Ferro” Using Morphometrics and Ecological Niche Modeling.” Brazilian Journal of Botany 47: 1–1219. 10.1007/s40415-024-01011-0.

[emi470157-bib-0056] Posit team . 2023. RStudio: Integrated Development Environment for R. Posit Software, PBC. http://www.posit.co/.

[emi470157-bib-0057] R Core Team . 2023. R: A Language and Environment for Statistical Computing. R Foundation for Statistical Computing. https://www.R‐project.org/.

[emi470157-bib-0058] Rafferty, N. E. , P. J. CaraDonna , and J. L. Bronstein . 2015. “Phenological Shifts and the Fate of Mutualisms.” Oikos 124, no. 1: 14–21. 10.1111/oik.01523. 25883391 PMC4396844

[emi470157-bib-0059] Rambaut, A. 2016. “Figtree v. 1.4.3.” http://tree.bio.ed.ac.uk/software/figtree/2016.

[emi470157-bib-0060] Reuter, M. , G. Bell , and D. Greig . 2007. “Increased Outbreeding in Yeast in Response to Dispersal by an Insect Vector.” Current Biology 17, no. 3: R81–R83. 10.1016/j.cub.2006.11.059. 17276903

[emi470157-bib-0061] Rivera, F. N. , E. Gonzalez , Z. Gomez , et al. 2009. “Gut‐Associated Yeast in Bark Beetles of the Genus *Dendroctonus* Erichson (Coleoptera: Curculionidae: Scolytinae).” Biological Journal of the Linnean Society 98, no. 2: 325–342. 10.1111/j.1095-8312.2009.01289.x.

[emi470157-bib-0062] Sampaio, J. P. , M. Gadanho , S. Santos , et al. 2001. “Polyphasic Taxonomy of the Basidiomycetous Yeast Genus *Rhodosporidium*: *Rhodosporidium kratochvilovae* and Related Anamorphic Species.” International Journal of Systematic and Evolutionary Microbiology 51, no. 2: 687–697. 10.1099/00207713-51-2-687. 11321116

[emi470157-bib-0063] Scheidler, N. H. , C. Liu , K. A. Hamby , F. G. Zalom , and Z. Syed . 2015. “Volatile Codes: Correlation of Olfactory Signals and Reception in *Drosophila*‐Yeast Chemical Communication.” Scientific Reports 5, no. 1: 14059. 10.1038/srep14059. 26391997 PMC4585764

[emi470157-bib-0064] Shen, S. K. , and P. F. Dowd . 1989. “Xenobiotic Induction of Esterases in Cultures of the Yeast‐Like Symbiont From the Cigarette Beetle.” Entomologia Experimentalis et Applicata 52, no. 2: 179–184. 10.1111/j.1570-7458.1989.tb01265.x.

[emi470157-bib-0065] Shen, S. K. , and P. F. Dowd . 1991. “Detoxification Spectrum of the Cigarette Beetle Symbiont *Symbiotaphrina kochii* in Culture.” Entomologia Experimentalis et Applicata 60, no. 1: 51–59. 10.1111/j.1570-7458.1991.tb01522.x.

[emi470157-bib-0066] Soto‐Robles, L. V. , V. Torres‐Banda , F. N. Rivera‐Orduña , E. Curiel‐Quesada , M. E. Hidalgo‐Lara , and G. Zúñiga . 2019. “An Overview of Genes From *Cyberlindnera americana*, a Symbiont Yeast Isolated From the Gut of the Bark Beetle *Dendroctonus rhizophagus* (Curculionidae: Scolytinae), Involved in the Detoxification Process Using Genome and Transcriptome Data.” Frontiers in Microbiology 10: 2180. 10.3389/fmicb.2019.02180. 31611850 PMC6777644

[emi470157-bib-0067] Starmer, W. T. , and J. C. Fogleman . 1986. “Coadaptation of *Drosophila* and Yeasts in Their Natural Habitat.” Journal of Chemical Ecology 12: 1037–1055. 10.1007/BF01638995. 24307046

[emi470157-bib-0068] Starmer, W. T. , and M. A. Lachance . 2011. “Yeast Ecology.” In The Yeasts, 65–83. Elsevier.

[emi470157-bib-0069] Stefanini, I. 2018. “Yeast‐Insect Associations: It Takes Guts.” Yeast 35, no. 4: 315–330. 10.1002/yea.3309. 29363168 PMC5947625

[emi470157-bib-0070] Stefanini, I. , L. Dapporto , L. Berná , M. Polsinelli , S. Turillazzi , and D. Cavalieri . 2016. “Social Wasps Are a *Saccharomyces* Mating Nest.” Proceedings of the National Academy of Sciences of the United States of America 113: 2247–2251. 10.1073/pnas.1516453113. 26787874 PMC4776513

[emi470157-bib-0071] Suesdek, L. , and F. C. T. Lima . 2011. “Association of the Borer *Spermophthorus apuleiae* (Coleoptera; Curculionidae; Scolytinae) With the “Pau‐Ferro” Tree *Caesalpinia Ferrea* (Leguminosae).” Biota Neotropica 11: 21–23. 10.1590/S1676-06032011000200001.

[emi470157-bib-0072] Suh, S. O. , and M. Blackwell . 2004. “Three New Beetle‐Associated Yeast Species in the *Pichia guilliermondii* Clade.” FEMS Yeast Research 5, no. 1: 87–95. 10.1016/j.femsyr.2004.06.001. 15381126

[emi470157-bib-0073] Suh, S. O. , and M. Blackwell . 2005. “Four New Yeasts in the *Candida mesenterica* Clade Associated With Basidiocarp‐Feeding Beetles.” Mycologia 97, no. 1: 167–177. 10.1080/15572536.2006.11832850. 16389968

[emi470157-bib-0074] Suh, S. O. , J. V. McHugh , D. D. Pollock , and M. Backwell . 2005. “The Beetle Gut: A Hyperdiverse Source of Novel Yeasts.” Mycological Research 109, no. 3: 261–265. 10.1017/S0953756205002388. 15912941 PMC2943959

[emi470157-bib-0075] Toki, W. 2021. “A Single Case Study of Mycetangia‐Associated Fungi and Their Abilities to Assimilate Wood‐Associated Carbon Sources in the Ship Timber Beetle *Elateroides flabellicornis* (Coleoptera: Lymexylidae) in Japan.” Symbiosis 83, no. 2: 173–181. 10.1007/s13199-021-00745-9.

[emi470157-bib-0076] Toki, W. 2023. “Fungal Community of Mycetangia in the Ship‐Timber Beetle *Elateroides dermestoides* (Coleoptera: Lymexylidae) in Japan.” Symbiosis 89: 299–305. 10.1007/s13199-023-00900-4.

[emi470157-bib-0077] Toki, W. , M. Tanahashi , K. Togashi , and T. Fukatsu . 2012. “Fungal Farming in a Non‐Social Beetle.” PLoS One 7, no. 7: e41893. 10.1371/journal.pone.0041893. 22848648 PMC3407107

[emi470157-bib-0078] Torto, B. , R. T. Arbogast , H. Alborn , et al. 2007. “Composition of Volatiles From Fermenting Pollen Dough and Attractiveness to the Small Hive Beetle *Aethina tumida*, a Parasite of the Honeybee *Apis mellifera* .” Apidologie 38, no. 4: 380–389. 10.1051/apido:2007024.

[emi470157-bib-0079] Ueda, H. , Y. Tachibana , M. Moriyasu , K. Kawanishi , and S. M. Alves . 2001. “Aldose Reductase Inhibitors From the Fruits of *Caesalpinia ferrea* Mart.” Phytomedicine 8, no. 5: 377–381. 10.1078/0944-7113-00043. 11695881

[emi470157-bib-0080] Urbina, H. , J. Schuster , and M. Blackwell . 2013. “The Gut of Guatemalan Passalid Beetles: A Habitat Colonized by Cellobiose‐ and Xylose‐Fermenting Yeasts.” Fungal Ecology 6, no. 5: 339–355. 10.1016/j.funeco.2013.06.005.

[emi470157-bib-0081] Vega, F. E. , and P. H. Biedermann . 2020. “On Interactions, Associations, Mycetangia, Mutualists and Symbiotes in Insect‐Fungus Symbioses.” Fungal Ecology 44: 100909. 10.1016/j.funeco.2019.100909.

[emi470157-bib-0082] White, T. J. , T. Bruns , S. J. W. T. Lee , and J. Taylor . 1990. “Amplification and Direct Sequencing of Fungal Ribosomal RNA Genes for Phylogenetics.” PCR Protocols: A Guide to Methods and Applications 18, no. 1: 315–322. 10.1016/B978-0-12-372180-8.50042-1.

[emi470157-bib-0083] Wickham, H. 2016. Ggplot2: Elegant Graphics for Data Analysis, 978‐3‐319‐24277‐4. Springer. https://ggplot2.tidyverse.org.

[emi470157-bib-0084] Witzgall, P. , M. Proffit , E. Rozpedowska , et al. 2012. ““This Is Not an Apple”–Yeast Mutualism in Codling Moth.” Journal of Chemical Ecology 38: 949–957. 10.1007/s10886-012-0158-y. 22797850

[emi470157-bib-0085] Wood, S. L. 1982. “The Bark and Ambrosia Beetles of North and Central America (Coleoptera: Scolytidae), a Taxonomic Monograph.” Great Basin Naturalist Memoirs 6: 1–1356.

[emi470157-bib-0086] Wood, S. L. 2007. Bark and Ambrosia Beetles of South America (Coleoptera, Scolytidae). Monte L. Bean Life Science Museum, Brigham Young University.

[emi470157-bib-0087] Yamamoto, D. , and W. Toki . 2023. “Presence of Non‐Symbiotic Yeasts in a Symbiont‐Transferring Organ of a Stag Beetle That Lacks Yeast Symbionts Found in Other Stag Beetles.” Scientific Reports 13, no. 1: 3726. 10.1038/s41598-023-30607-x. 36918653 PMC10014939

