# Supplementary material for: When Two Species Meet: A Potential Beetle‐Yeast Facultative Mutualism
Source: Environ Microbiol Rep. 2025 Jul 14;17(4):e70157. doi: 10.1111/1758-2229.70157 (PMC12259496; doi:10.1111/1758-2229.70157)
Supplement: Supplementary file 1 — Data S1. [file EMI4-17-e70157-s001.docx]

When two species meet: a potential beetle-yeast facultative mutualism

Rodolfo Bizarria Jr., Tatiane de Castro Pietrobon, Pepijn W. Kooij, Andre Rodrigues

**Supplementary Material**

Climate Data and Yeast Count Analysis. We graphically compared the fruiting periods of *Libidibia ferrea* (from May to December; Carvalho, 2003) with the climatic conditions in the state of São Paulo. We used the averages of temperature (average, minimum, and maximum) and precipitation between the periods 2019 to 2021. We obtained available monthly climate data from the Integrated Center for Agrometeorological Information of the State of São Paulo ([www.ciiagro.org.br](http://www.ciiagro.org.br)), throughout five cities of the state (Araras-SP, Limeira-SP, Mogi-Guaçu-SP, Piracicaba-SP, and Rio Claro-SP). To compare the yeast colony-forming units with environmental conditions, we retrieved daily climatic data (maximum and minimum temperature, precipitation, and humidity) of São Paulo cities from the seven days prior to each fruit sampling. For this purpose, data were retrieved from INMET, the Brazilian National Institute of Meteorology, and CIIAGRO. We chose the stations based on data availability for the period and their proximity to the sampling points. We calculated the averages of each climatic variable, except for precipitation, where we used the sum. To analyze whether the effects of temperature, humidity, and precipitation, preceding the fruit collection by seven days, influence the yeast colony-forming units, we initially assessed the climatic data for normality using the Shapiro-Wilk normality test, and since none of the variables followed a normal distribution, we conducted the Spearman's rank correlation tests to assess the relationships between climatic variables and yeast count data. However, only precipitation (rho = 0.647, p < 0.001) and humidity (rho = 0.745, p < 0.001) showed a significant and positive correlation with yeast counts, indicating that these factors may influence the response variable. Since temperature did not showed a significant correlation it was not included in the following analyses.

To explore the effects of climatic data on yeast counts, we fitted a generalized linear mixed model using a Negative Binomial distribution. The model included the number of yeast colony-forming units as the response variable and precipitation and humidity as fixed effects. To validate the model, we inspected the overdispersion, residuals, and the presence of zero inflation using the R-package DHARMa (Hartig, 2022). Our results indicated that humidity had a highly significant effect (Estimate = 0.292, p < 0.01), while precipitation and its interaction with humidity were marginally significant (Precipitation: Estimate = 0.361, p = 0.056; Interaction: Estimate = -0.005, p = 0.069; Table S10). To further explore the significance of the predictors, we performed an analysis of deviance which indicates that precipitation, humidity and their interaction significatly improves the model fit (Table S10). Overall the results indicate that both precipitation and humidity significantly affect the yeast counts response, with evidence of an interaction between the fixed effects. We conducted all analyses in RStudio v. 2023.12.0.369 (Posit team, 2023) using R v.4.3.2 (R Core Team, 2023), and ggplot2 package (Wickham, 2016) for plot creation.

Biodiversity Analysis. We described the yeast community found in infested fruits by calculating diversity metrics using the diversityresult function from the “BiodiversityR” library (Kindt and Coe, 2005), the specpool function from the “vegan” library (Oksanen et al., 2024), and software EstimateS v. 9.1.0 (Colwell, 2013). We computed rarefaction curves for species richness with the “iNEXT” library (Chao et al., 2014; Hsieh and Chao, 2024) using abundance data. A total of 31 infested fruit samples were examined, revealing a species richness of 12 and an abundance of 598 isolates. The diversity indices, such as Shannon, Simpson, and Inverse Simpson, indicate moderate diversity within the samples. The Jevenness and Eevenness indices highlight the uneven distribution of individuals among species, suggesting that certain species, such as *Cyberlindnera* sp., are much more dominant. Furthermore, richness estimators like Jackknife and Chao suggest the potential for undiscovered species within the samples. The higher values of these richness estimators imply that additional sampling could uncover more species, indicating that the current sampling may not have fully captured the diversity of the infested fruits (also supported by the observed pattern of the rarefaction curve), an environment notably dominated by *Cyberlindnera* sp.

To compare the fruit samples, we standardized the species data with the vegdist function from the “vegan” library (Oksanen et al., 2024), setting Bray-Curtis distance. We performed clustering analyses using the hclust function from the “stats” library (R Core Team, 2023), using the complete method as an agglomeration criterion for hierarchical clustering. We calculated the cophenetic correlation coefficient (0.946) to ensure that the dendrogram adequately represented the species data, applying the cophenetic function from the “stats” library (R Core Team, 2023). By setting a Bray-curtis distance cut-off of 0.6, we identified seven distinct clusters, that include a mix of external and internal samples, and some groups with clear distinctions between the source of samples. Additionally, isolated groups were observed to contain samples that did not contain Cyberlindnera in their composition.

To determine whether internal or external yeast communities differ among the beetles, we performed a nonmetric multidimensional scaling (nMDS) analysis using the metaMDS function from the “vegan” library (Oksanen et al., 2024) and principal coordinates analysis (PCoA) using the cmdscale function from the “stats” library (R Core Team, 2023), in both cases with Bray–Curtis distances. In the nMDS analysis, we removed an external sample from a male beetle due to discrepancies in the MDS1 and MDS2 values. We conducted analyses of variance to test if differences in yeast communities are based on the yeast source (internal or external) and/or beetle sex (males or females). We performed an analysis of variance using the distance matrices with the adonis2 function from the “vegan” library (Oksanen et al., 2024). We found that differences in community composition between beetle sexes were not statistically significant (DF = 1, p-value = 0.821), while the source of isolation (internal or external) had a significant effect on yeast community composition (DF = 1, p-value = 0.001). The differences between the sources of isolation were further supported by nMDS and PCoA analyses. In the nMDS analysis, the first dimension explained 56.87% of the variation, while the second dimension accounted for 43.13%, with a stress value of 0.0464, indicating a good fit. In the PCoA analysis, Axis 1 explained 43.4% of the variation, and Axis 2 explained 21.6%, highlighting distinct clustering based on the source of isolation. We conducted all analyses in RStudio v. 2023.12.0.369 (Posit team, 2023) using R v.4.3.2 (R Core Team, 2023), and ggplot2 package (Wickham, 2016) for plot creation.

**Figures**

**Figure S1.** Infested fruits are an environment with *Cyberlindnera* sp. dominance, and differences between beetle internal and external yeast community were observed. **A.** Rarefaction curve of yeast species from fruit samples, with the potential for undiscovered species as indicated by dashed lines. **B.** Hierarchical clustering highlighting the differences between internal and external samples, as well as by groups that lack *Cyberlindnera*. **C.** Principal Coordinates Analysis (PCoA) and **D.** Nonmetric Multidimensional Scaling (nMDS) indicating the differences between internal and external yeast community composition.

**
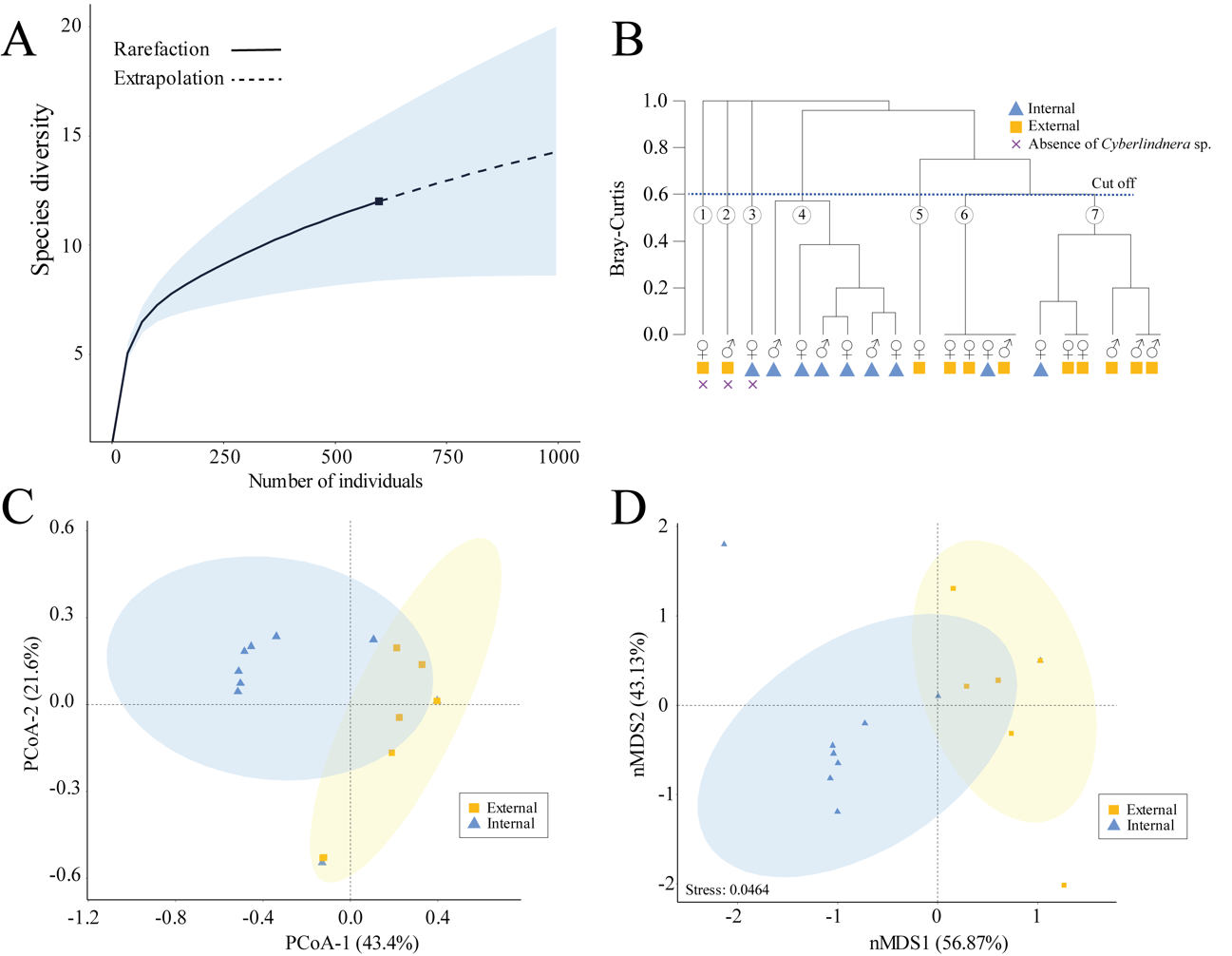
**

**Figure S2.** Phylogeny of *Pseudozyma hubeiensis* (B5, in bold) using D1/D2 and ITS as partitions. Tree inferred with maximum likelihood criteria. Numbers on branches are ultrafast bootstrap support values (only values higher than 70). Phylogeny statistics were provided in Table S2. The scale bar denotes substitutions per site.


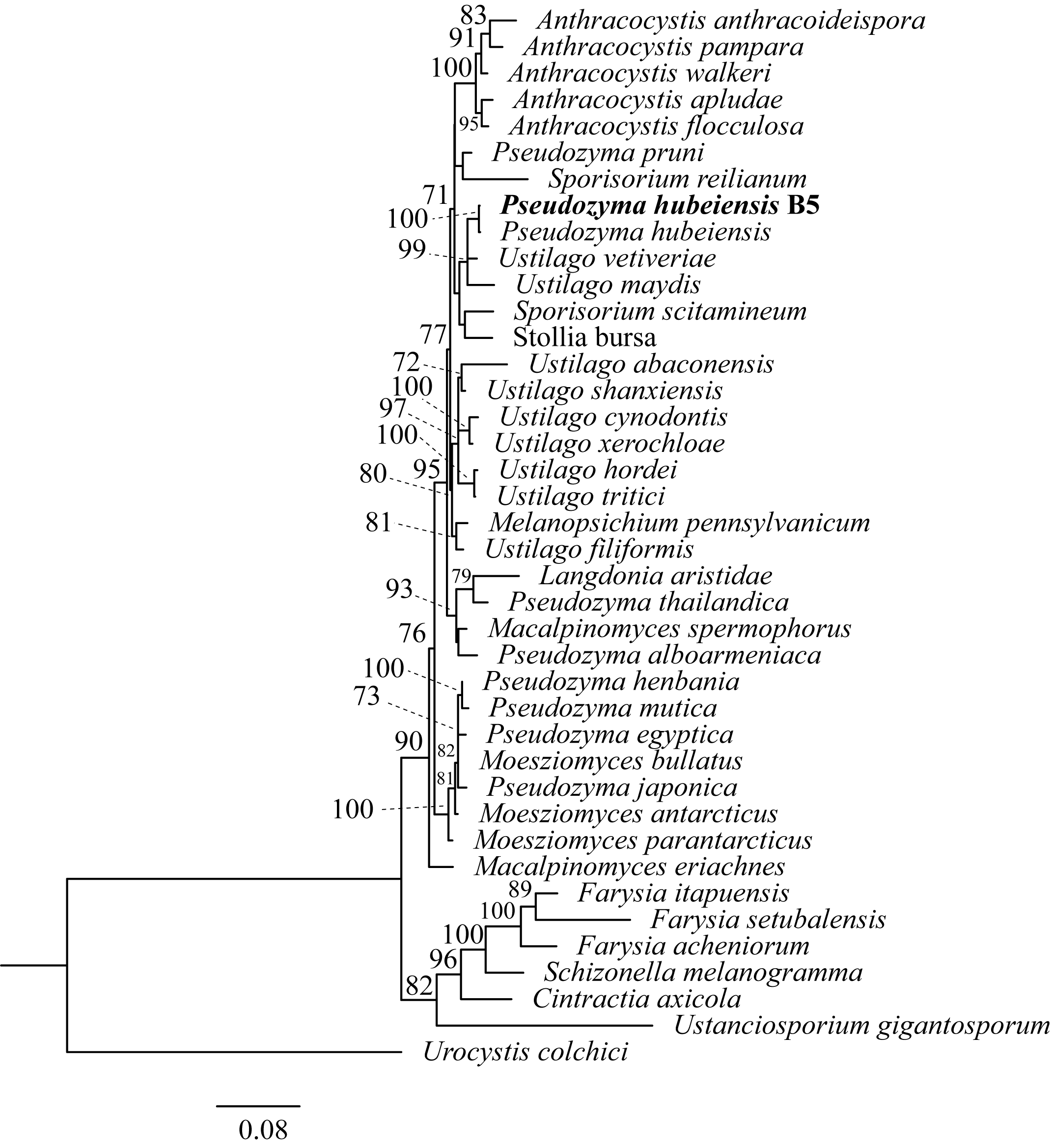


**Figure S3.** Phylogeny of *Ogataea* sp. (B127, in bold) using D1/D2 and ITS as partitions. Tree inferred with maximum likelihood criteria. Numbers on branches are ultrafast bootstrap support values (only values higher than 70). Phylogeny statistics were provided in Table S2. The scale bar denotes substitutions per site.

**
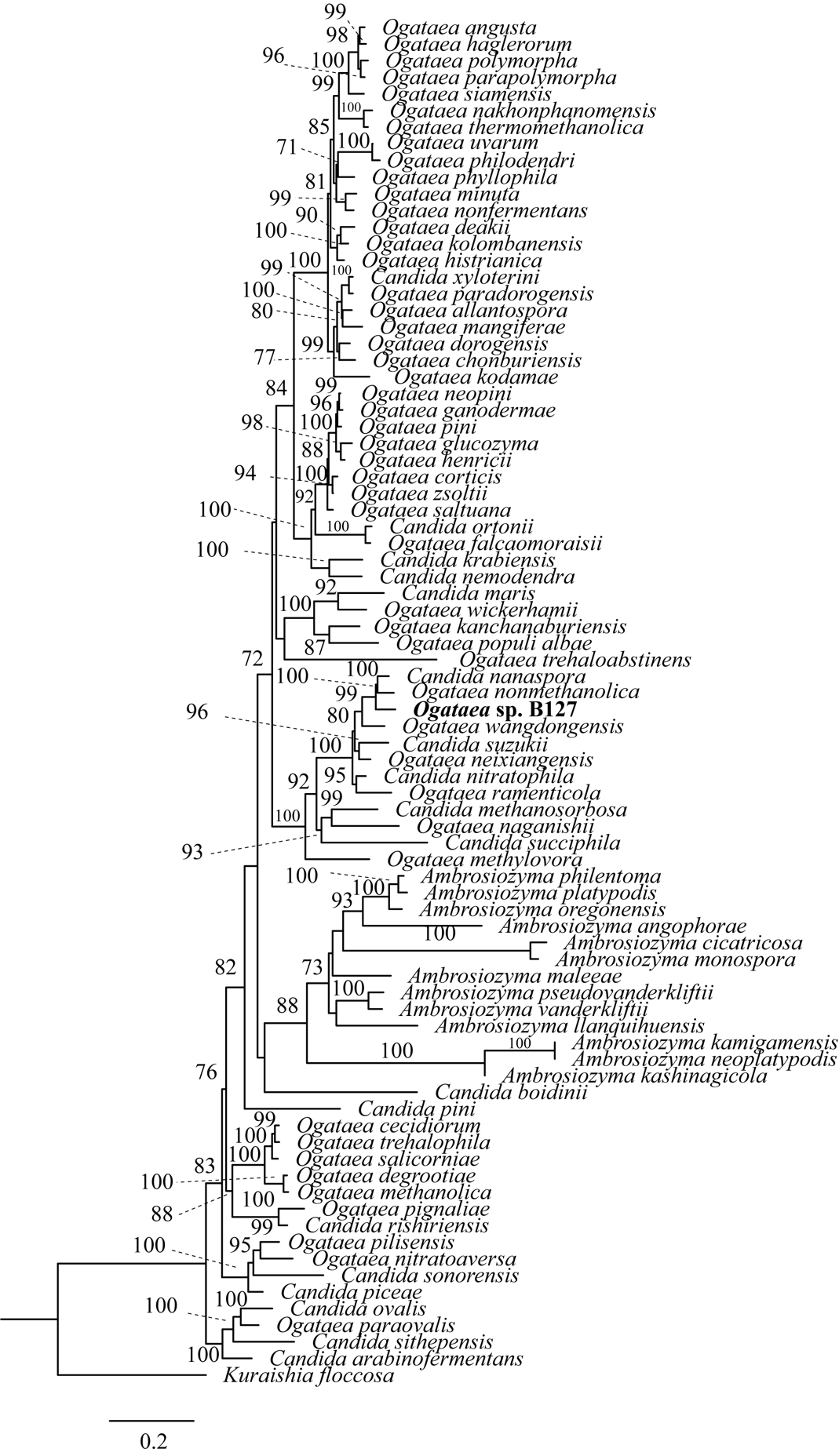
**

**Figure S4.** Phylogeny of *Yamadazyma riverae* (B343, in bold) using D1/D2 and ITS as partitions. Tree inferred with maximum likelihood criteria. Numbers on branches are ultrafast bootstrap support values (only values higher than 70). Phylogeny statistics were provided in Table S2. The scale bar denotes substitutions per site.

**
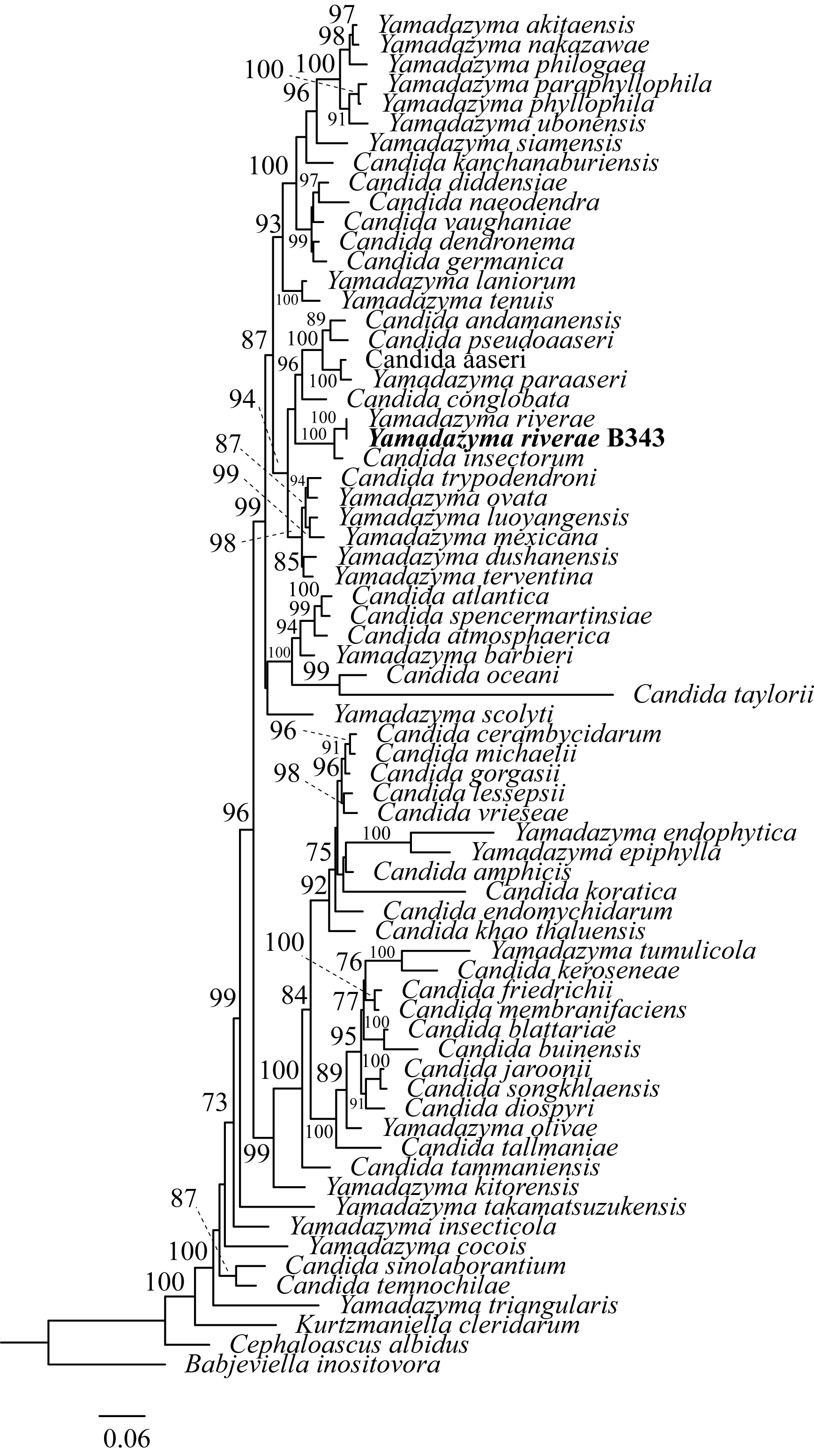
**

**Figure S5.** Phylogeny of *Aureobasidium* sp. (B437, in bold) using D1/D2 and ITS as partitions. Tree inferred with maximum likelihood criteria. Numbers on branches are ultrafast bootstrap support values (only values higher than 70). Phylogeny statistics were provided in Table S2. The scale bar denotes substitutions per site.

**
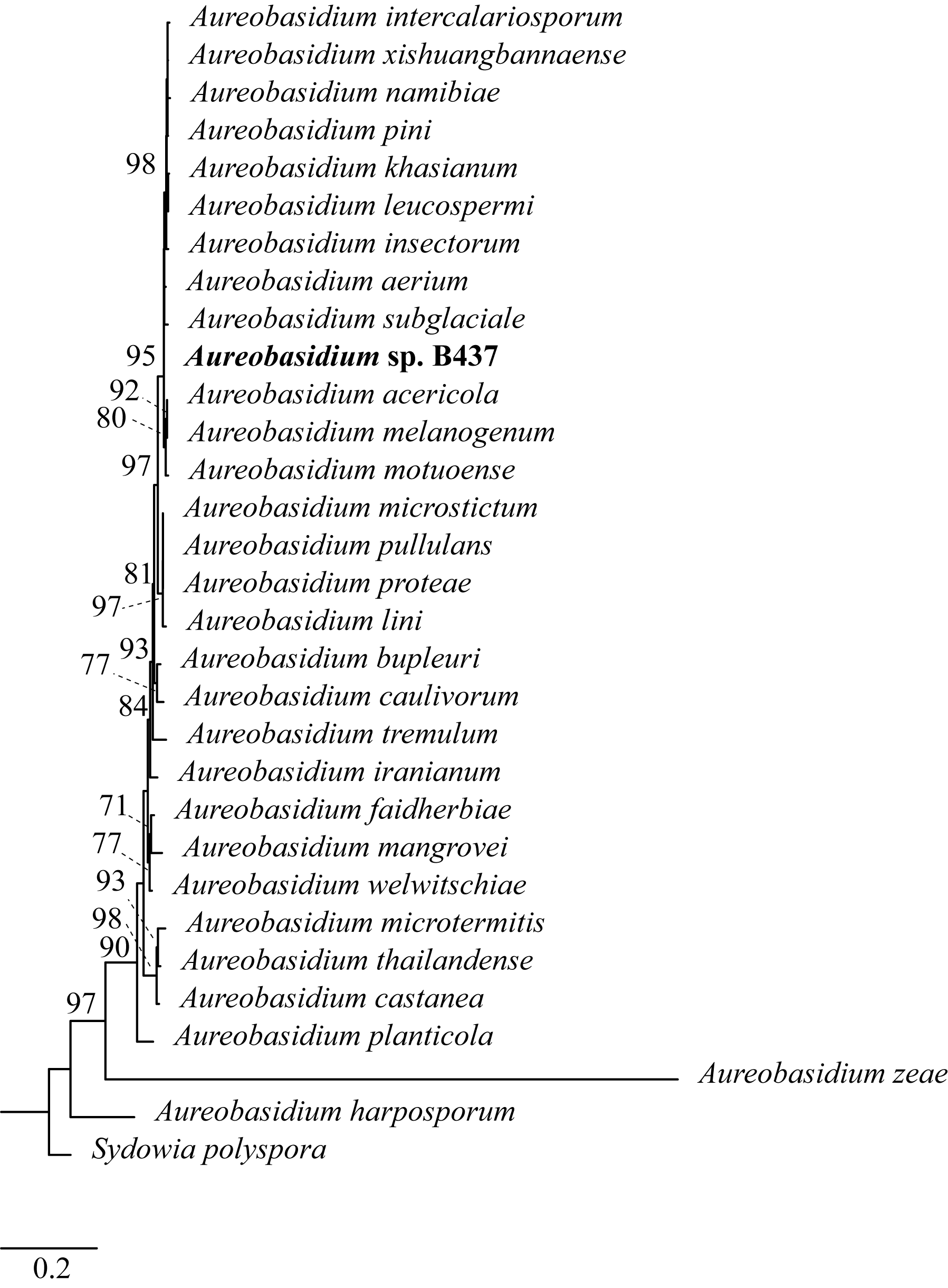
**

**Figure S6.** Phylogeny of *Curvibasidium* sp. (B455, in bold) using D1/D2 and ITS as partitions. Tree inferred with maximum likelihood criteria. Numbers on branches are ultrafast bootstrap support values (only values higher than 70). Phylogeny statistics were provided in Table S2. The scale bar denotes substitutions per site.

**
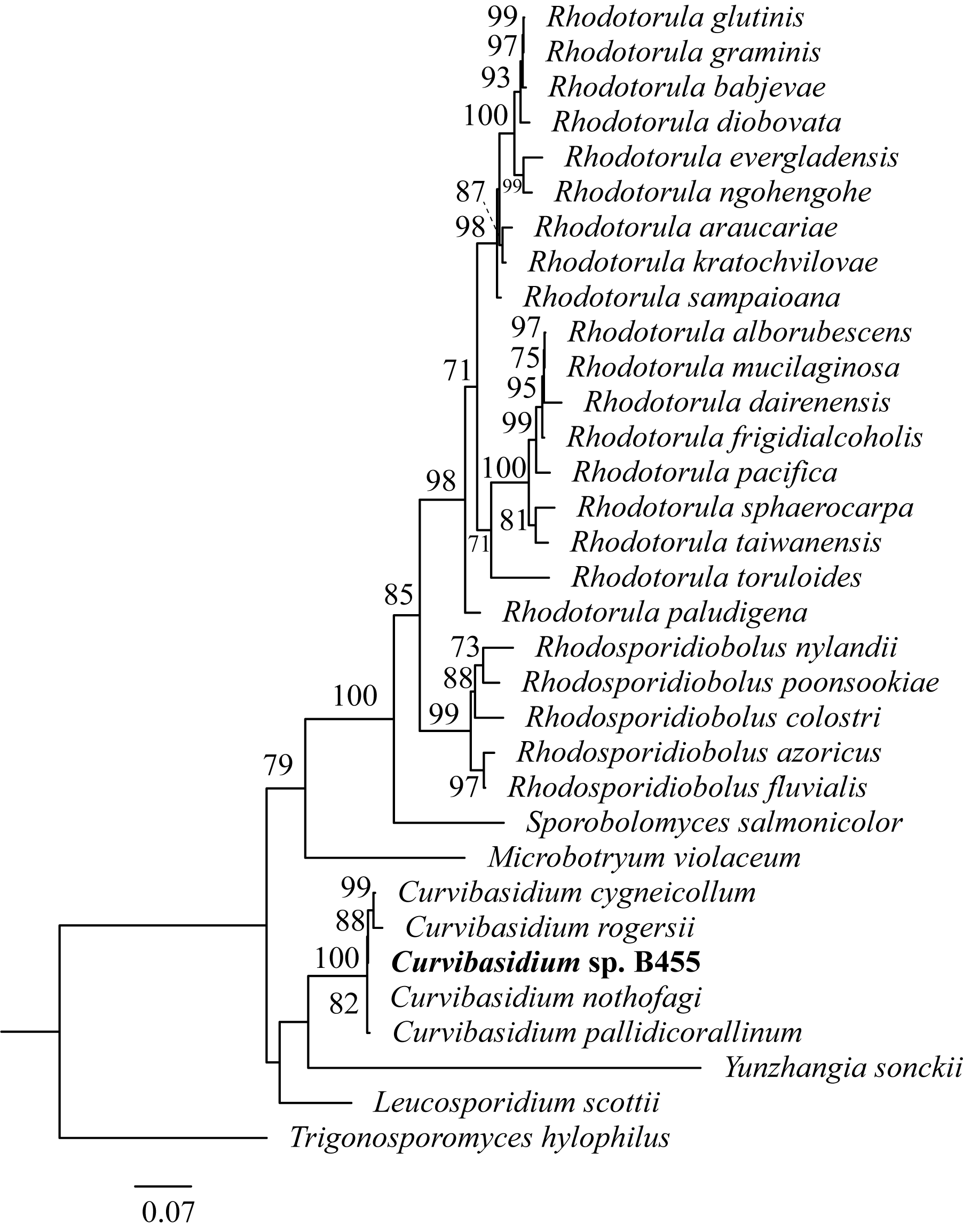
**

**Figure S7.** Phylogeny of *Zygoascus* sp. (B477, in bold) using D1/D2. Tree inferred with maximum likelihood criteria. Numbers on branches are ultrafast bootstrap support values (only values higher than 70). Phylogeny statistics were provided in Table S2. The scale bar denotes substitutions per site.


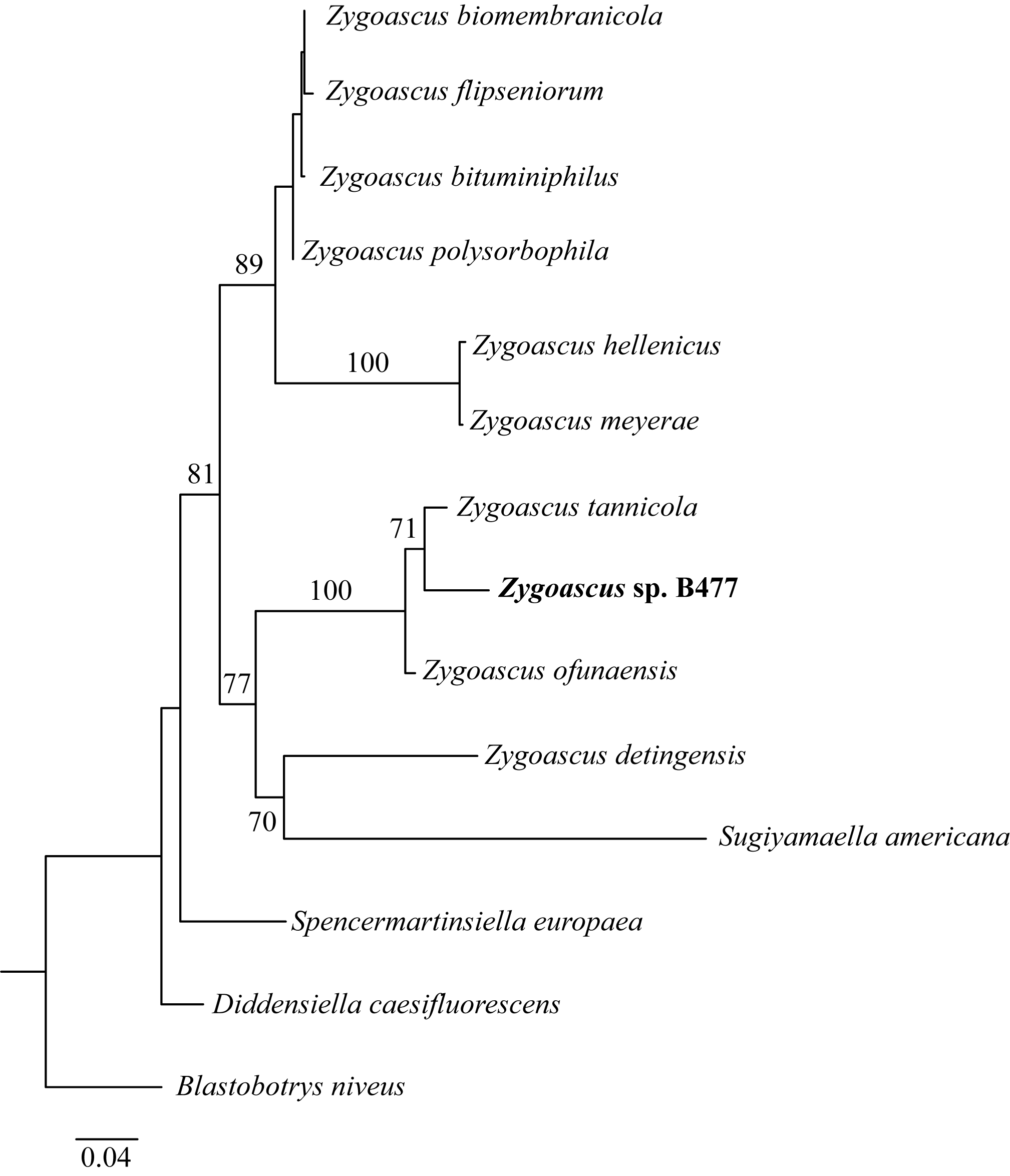


**Figure S8.** Phylogeny of *Meyerozyma caribbica* (B565, in bold) using D1/D2 and ITS as partitions. Tree inferred with maximum likelihood criteria. Numbers on branches are ultrafast bootstrap support values (only values higher than 70). Phylogeny statistics were provided in Table S2. The scale bar denotes substitutions per site.

**
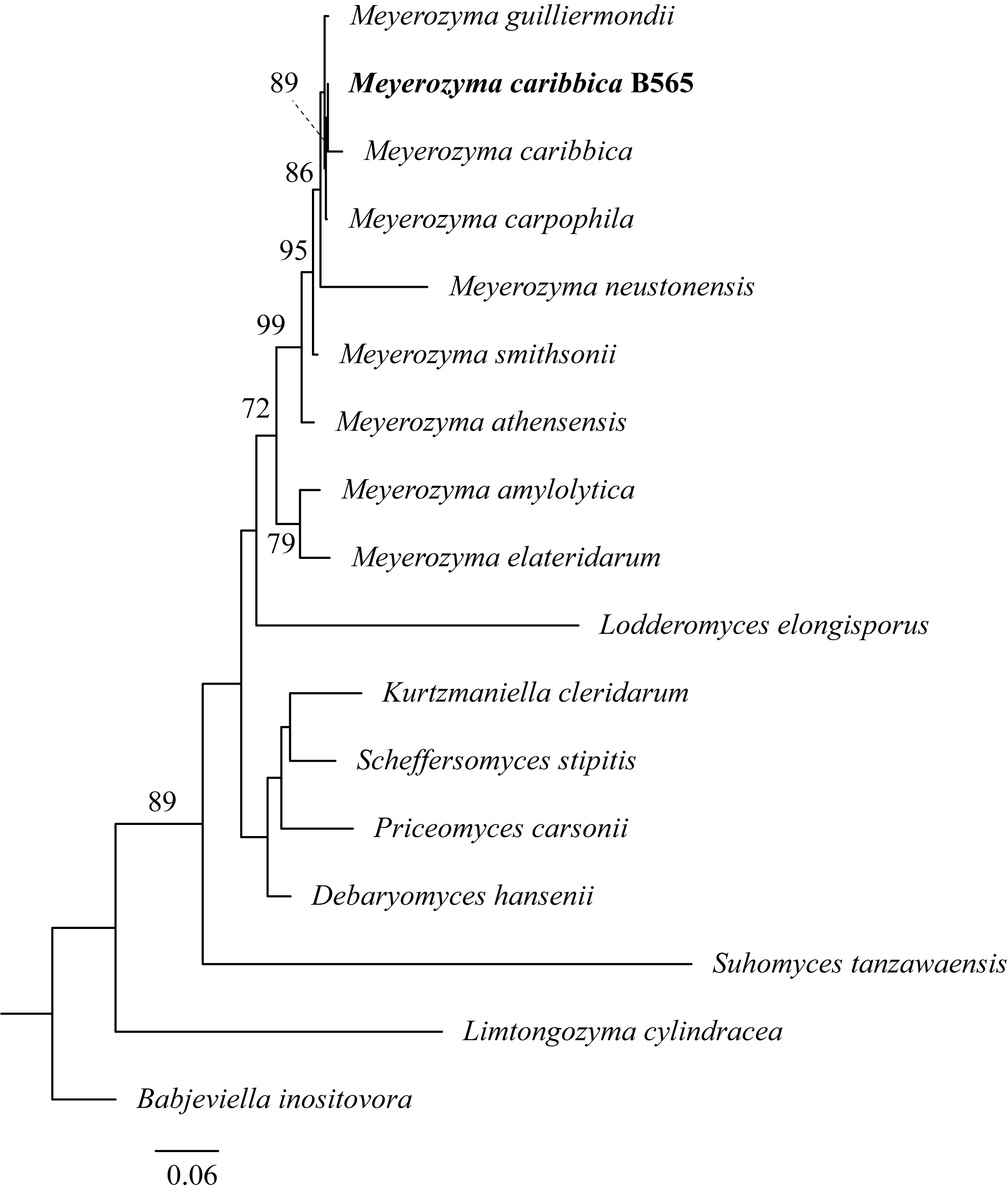
**

**Figure S9.** Phylogeny of *Kwoniella mangrovensis* (B587, in bold) and *Teunia* sp. (B641, in bold) using D1/D2 and ITS as partitions. Tree inferred with maximum likelihood criteria. Numbers on branches are ultrafast bootstrap support values (only values higher than 70). Phylogeny statistics were provided in Table S2. The scale bar denotes substitutions per site.

**
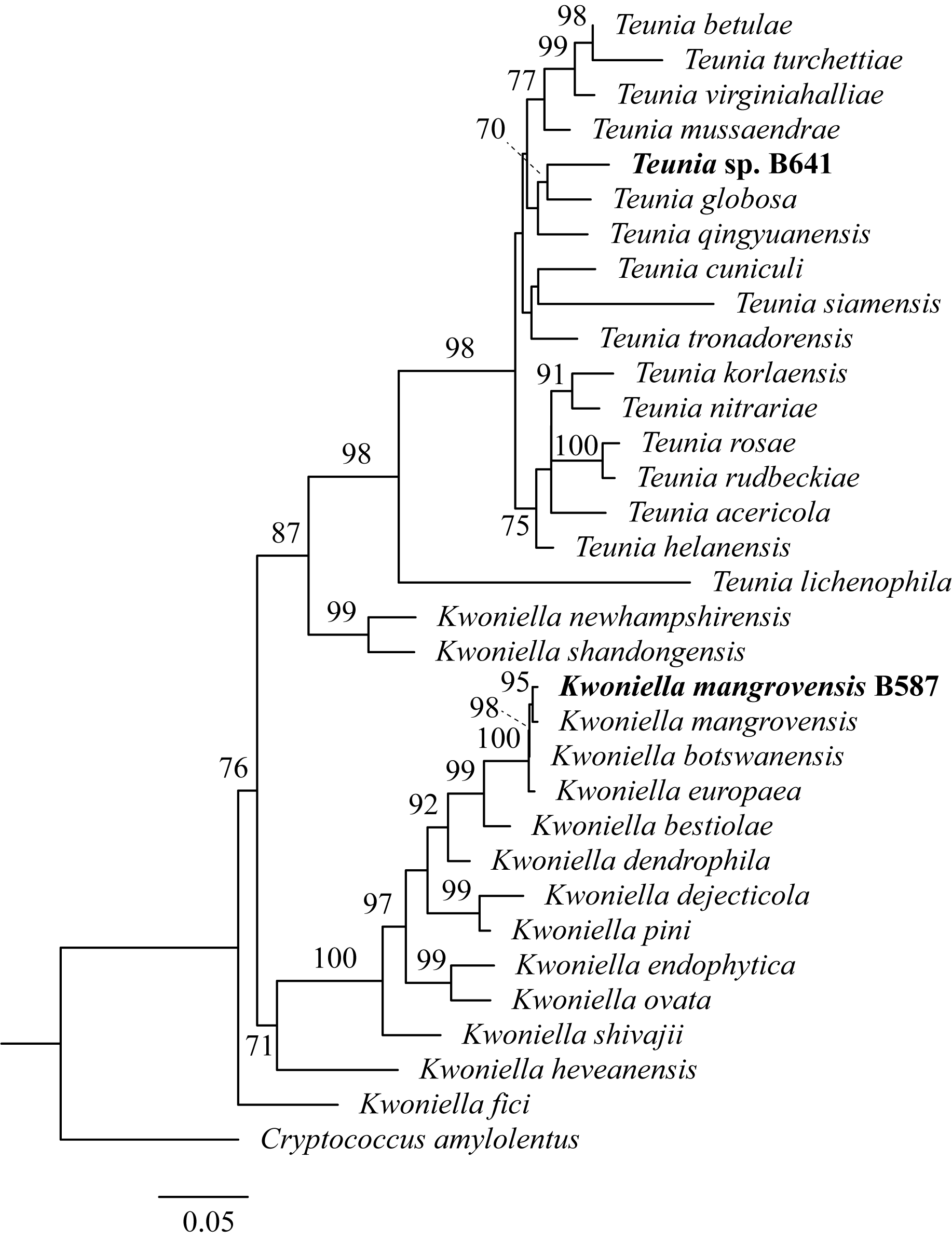
**

**Figure S10.** Phylogeny of *Cyberlindnera* sp. (B620, in bold) using D1/D2 and ITS as partitions. Tree inferred with maximum likelihood criteria. Numbers on branches are ultrafast bootstrap support values (only values higher than 70). Phylogeny statistics were provided in Table S2. The scale bar denotes substitutions per site.

**
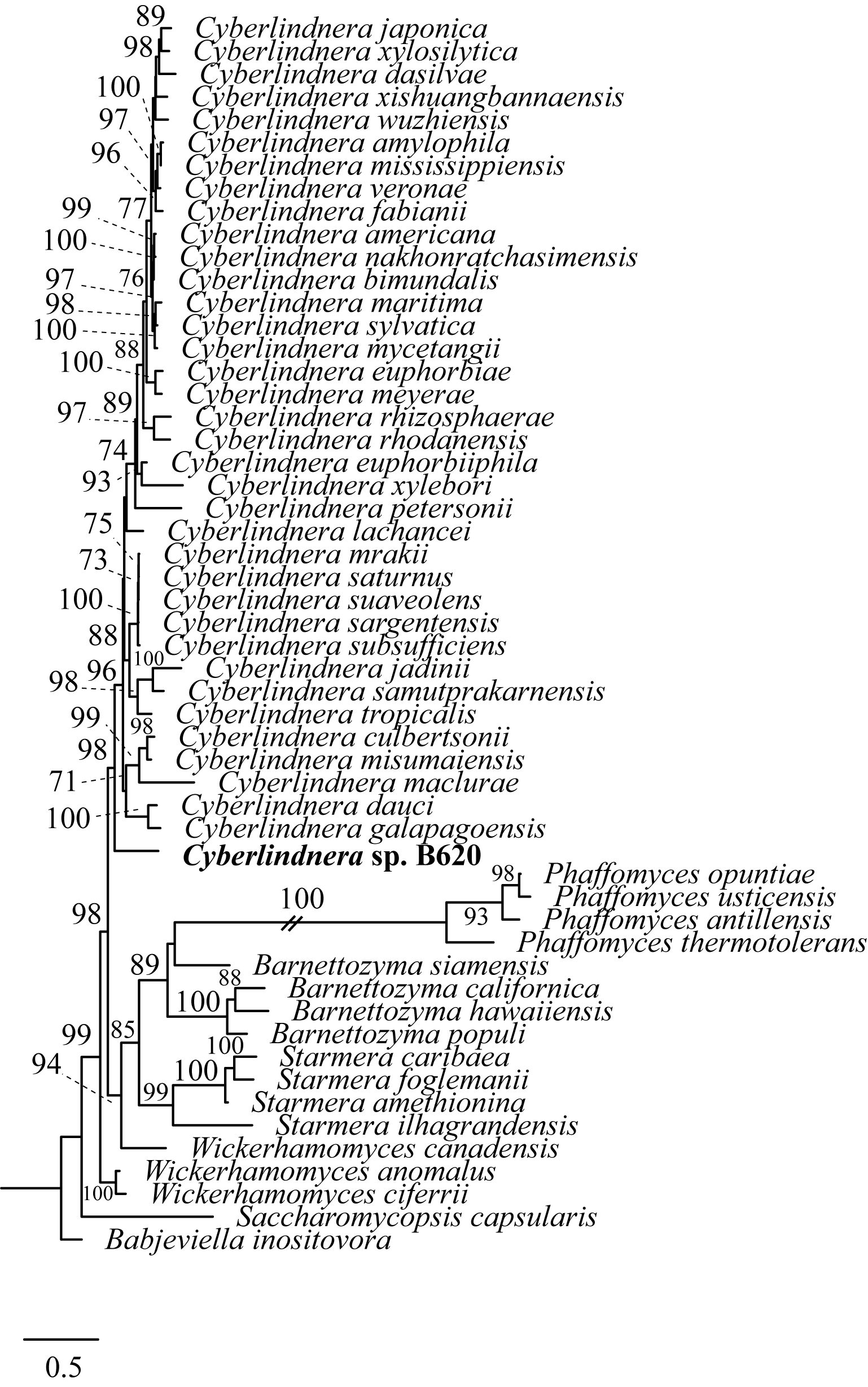
**

**Figure S11.** Phylogeny of *Sirobasidiaceae* sp. (B632, in bold) using D1/D2 and ITS as partitions. Tree inferred with maximum likelihood criteria. Numbers on branches are ultrafast bootstrap support values (only values higher than 70). Phylogeny statistics were provided in Table S2. The scale bar denotes substitutions per site.


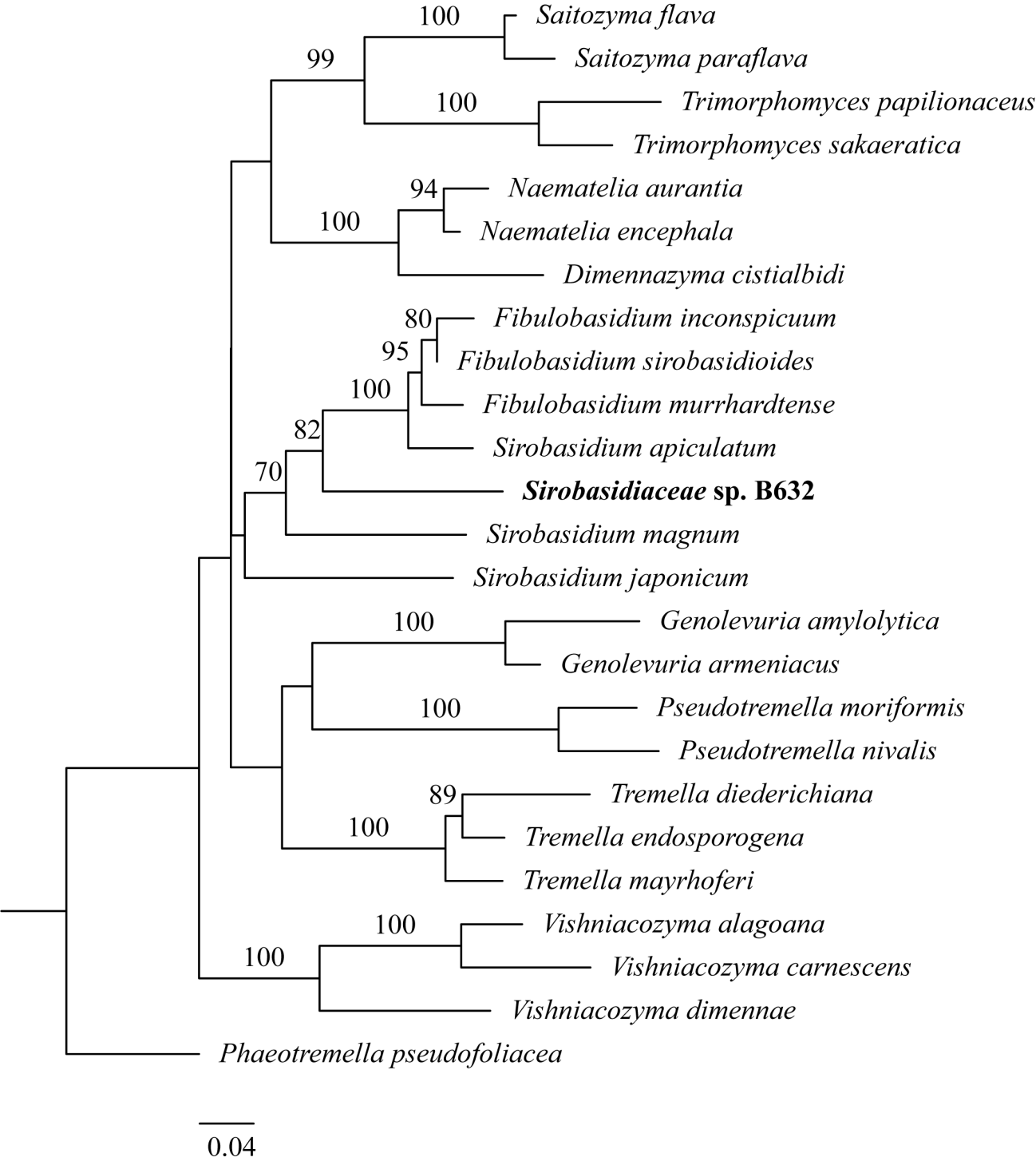


**Figure S12.** Phylogeny of *Jaminaea* sp. (B633, in bold) using D1/D2 and ITS as partitions. Tree inferred with maximum likelihood criteria. Numbers on branches are ultrafast bootstrap support values (only values higher than 70). Phylogeny statistics were provided in Table S2. The scale bar denotes substitutions per site.


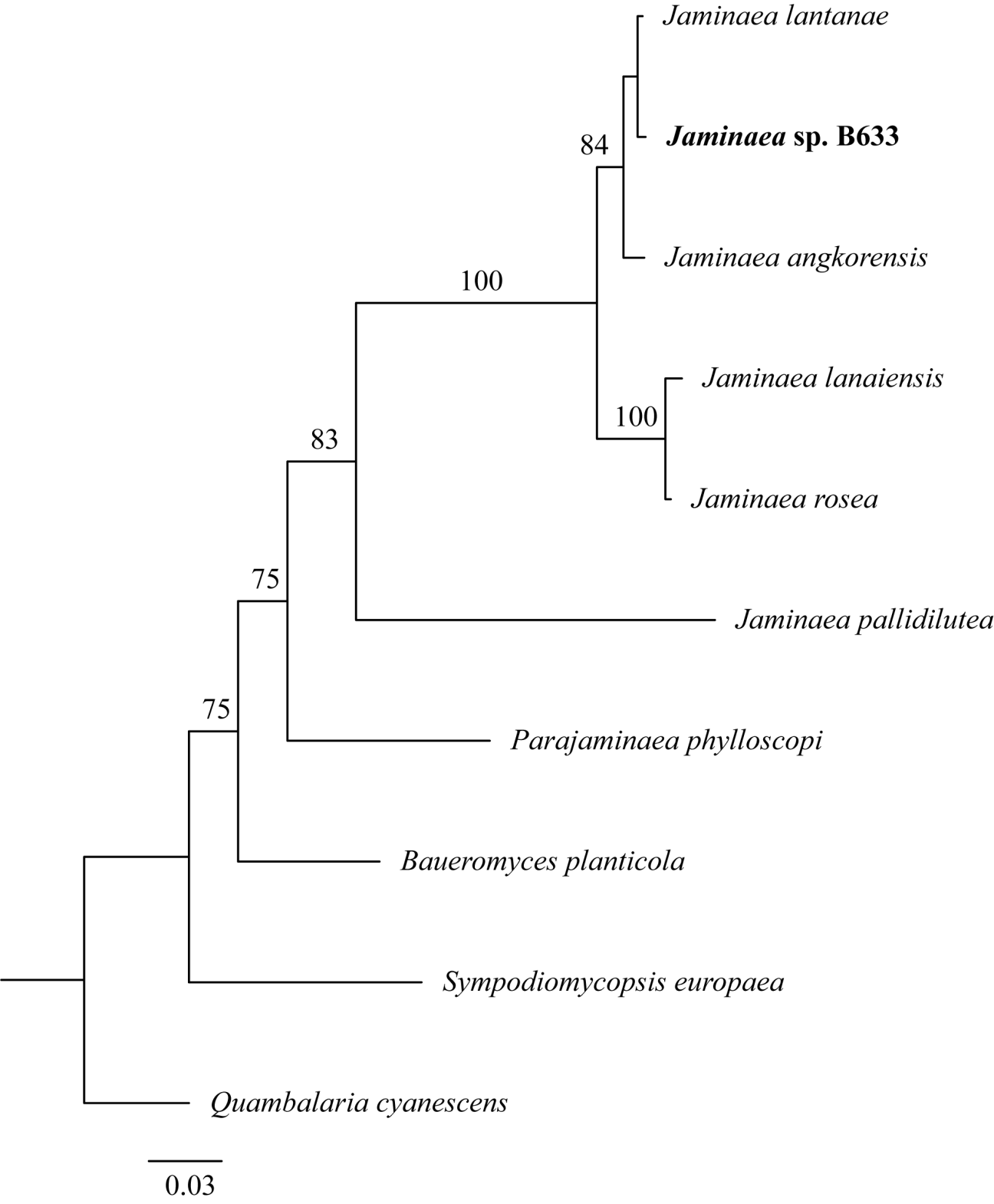


**Figure S13.** Phylogeny of *Pseudosydowia* sp. (B640, in bold) using D1/D2 and ITS as partitions. Tree inferred with maximum likelihood criteria. Numbers on branches are ultrafast bootstrap support values (only values higher than 70). Phylogeny statistics were provided in Table S2. The scale bar denotes substitutions per site.


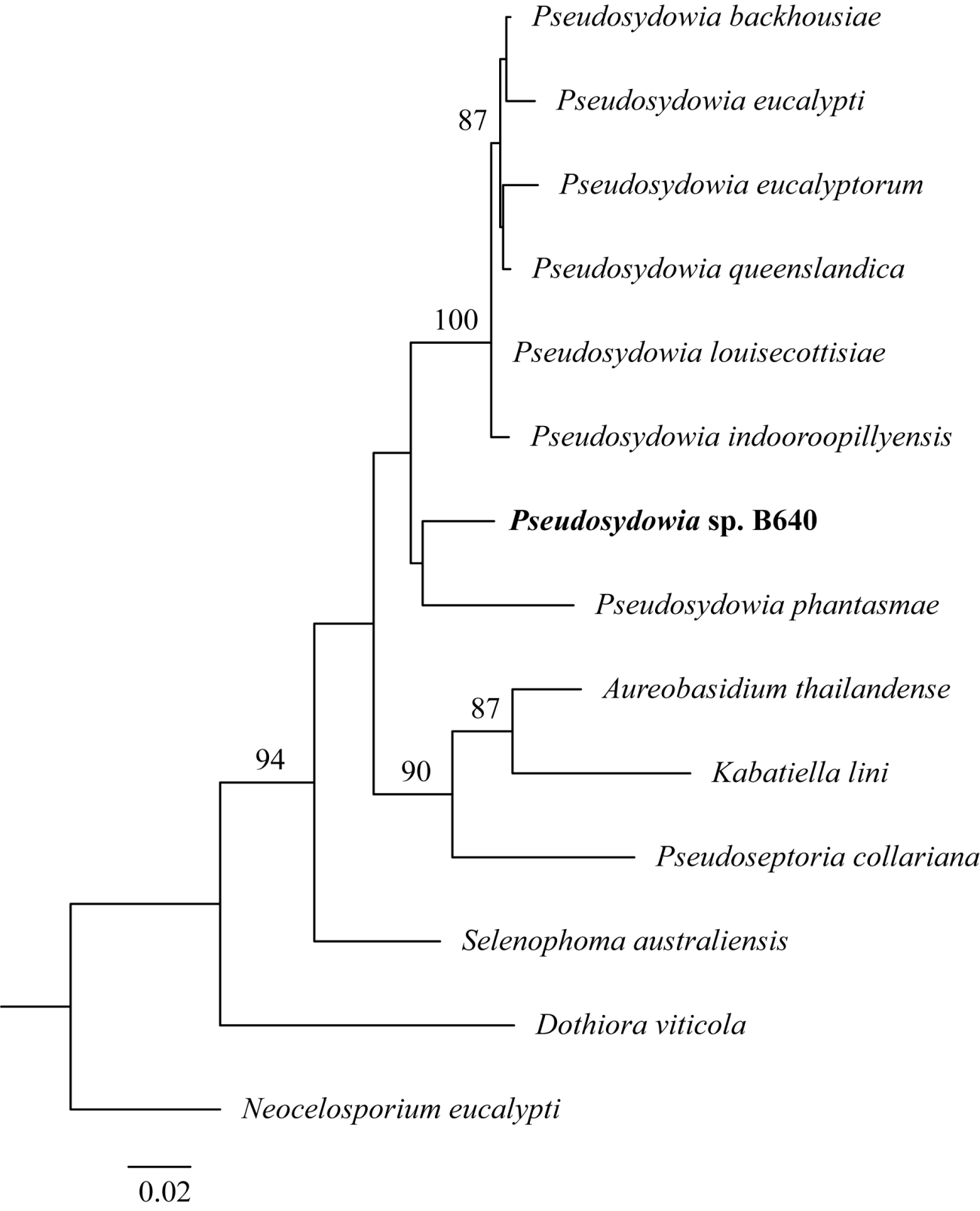


**Figure S14.** Phylogeny of *Candida railenensis* (B658, in bold) and *Candida oleophila* (B660, in bold) using D1/D2 and ITS as partitions. Tree inferred with maximum likelihood criteria. Numbers on branches are ultrafast bootstrap support values (only values higher than 70). Phylogeny statistics were provided in Table S2. The scale bar denotes substitutions per site.


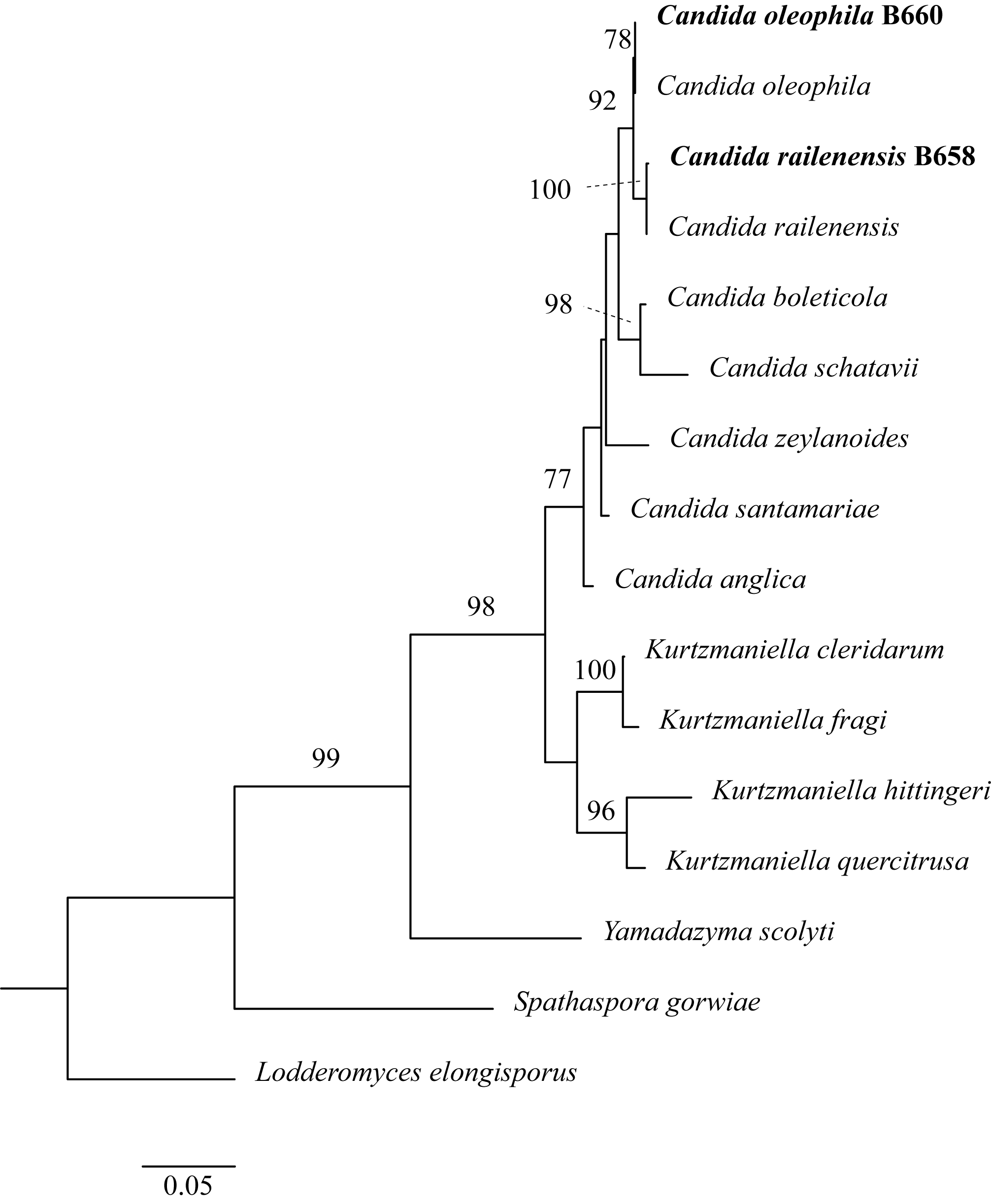


**Figure S15.** Graphical exploration of climate variables and yeast counts. **A.** Histogram of climate data and fruiting season periods of *Libidibia ferrea* (May to December). Months of fruit collection were highligned by triangles. **B.** Curve between precipitation and **C.** Humidity with a positive correlation with yeast counts.

**
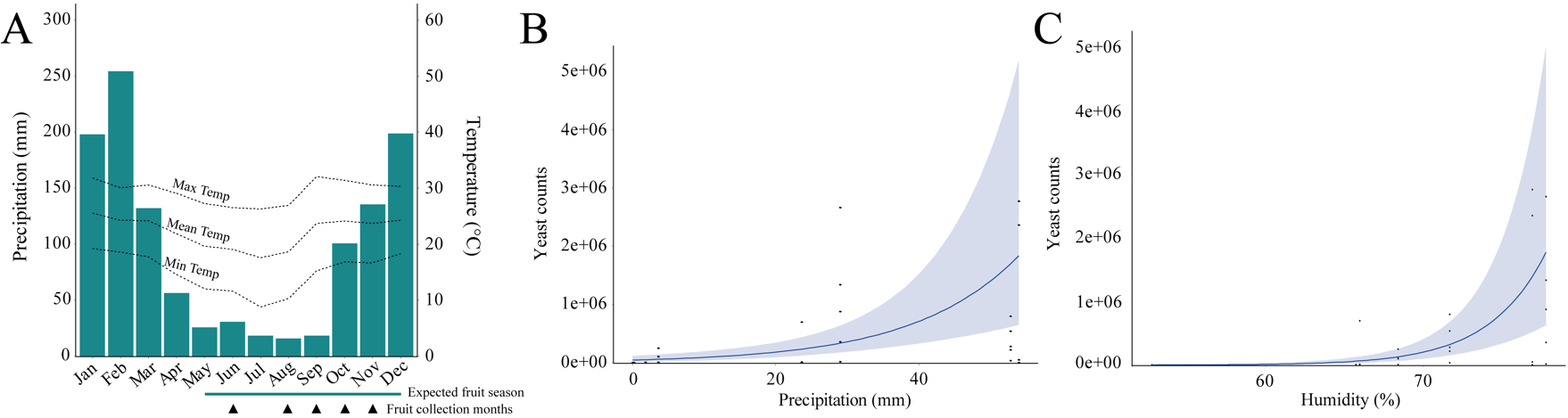
**

**Tables**

**Table S1.** Phylogeny and model generation statistics.

| **Strain ID** | **Species** | **Alignment (pb)** | **ITS/LSU model** | **Iterations/Log-likelihood** |
| --- | --- | --- | --- | --- |
| B5 | *Pseudozyma hubeiensis* | 1201 | SYM+I+R2 / K2P+I+R2 | 103 / -4745.466 |
| B127 / LESF1535 | *Ogataea* sp. | 1527 | GTR+F+I+G4 | 133 / -23049.014 |
| B343 / LESF1538 | *Yamadazyma riverae* | 1289 | GTR+F+I+G4/GTR+F+R3 | 137 / -13349.725 |
| B437 / LESF1872 | *Aureobasidium* sp. | 1201 | SYM+I+R2/K2P+I+R2 | 103 / -4745.466 |
| B455 / LESF1848 | *Curvibasidium* sp. | 1276 | GTR+F+I+G4/GTR+F+I+R2 | 102 / -8110.943 |
| B477 / LESF1510 | *Zygoascus* sp. | 1270 | GTR+F+G4 | 102 / -2569.610 |
| B565 / LESF1537 | *Meyerozyma caribbica* | 1236 | HKY+F+G4/GTR+F+I+R2 | 102 / -6295.320 |
| B587 / LESF1847 | *Kwoniella mangrovensis* | 1269 | SYM+I+G4/SYM+I+R2 | 102 / -7218.353 |
| B620 / LESF1555 | *Cyberlindnera* sp. | 1451 | GTR+F+I+G4 | 102 / -17888.453 |
| B632 / LESF1846 | *Sirobasidiaceae* sp. | 1269 | SYM+I+G4/K2P+I+G4 | 107 / -9500.063 |
| B633 / LESF1873 | *Jaminaea* sp. | 1306 | HKY+F+G4/K2P+I | 102 / -4314.734 |
| B640 / LESF1874 | *Pseudosydowia* sp. | 1208 | SYM+G4/ K2P+I | 102 / -4021.017 |
| B641 / LESF1850 | *Teunia* sp. | 1269 | SYM+I+G4/SYM+I+R2 | 102 / -7218.353 |
| B658 / LESF1845 | *Candida railenensis* | 1227 | GTR+F+G4 | 102 / -4495.479 |
| B660 / LESF1849 | *Candida oleophila* | 1227 | GTR+F+G4 | 102 / -4495.479 |

**Table S2.** Sequence data used in phylogenetic analyses.

| **Species** | **Strain** | **ITS** | **LSU** |
| --- | --- | --- | --- |
| *Ambrosiozyma angophorae* | CBS 5823/NRRL Y-7118 | KY101634 | EU011592 |
| *Ambrosiozyma cicatricosa* | CBS 6157/NRRL Y-17594 | KY101637 | EU011591 |
| *Ambrosiozyma kamigamensis* | CBS 10899 | KY101638 | KY106096 |
| *Ambrosiozyma kashinagicola* | CBS 10903/NRRL Y-63631 | KY101639 | KF061197 |
| *Ambrosiozyma llanquihuensis* | CBS 8182/NRRL Y-17657 | KY101640 | EU011589 |
| *Ambrosiozyma maleeae* | CBS 11900/NRRL Y-63635 | KY101641 | KF061198 |
| *Ambrosiozyma monospora* | CBS 2554/NRRL Y-1484 | KY101643 | EU011590 |
| *Ambrosiozyma neoplatypodis* | CBS 10900 | KY101645 | KY106102 |
| *Ambrosiozyma oregonensis* | CBS 5560 | KY101646 | KY106103 |
| *Ambrosiozyma philentoma* | CBS 6276/NRRL Y-7523 | KY101647 | EU011595 |
| *Ambrosiozyma platypodis* | CBS 4111/NRRL Y-6732 | KY101651 | EU011594 |
| *Ambrosiozyma pseudovanderkliftii* | CBS 10904/NRRL Y-63632 | KY101653 | KF061199 |
| *Ambrosiozyma vanderkliftii* | CBS 10905/NRRL Y-63633 | KY101654 | KF061200 |
| *Anthracocystis anthracoideispora* | HUV 18350 | JN367290 | JN367315 |
| *Anthracocystis apludae* | KVU 967 | JN367294 | JN367319 |
| *Anthracocystis flocculosa* | JCM10321/CBS:167.88 | AB089364 | AJ235299 |
| *Anthracocystis pampara* | JCM 2007 | KP322980 | KP322980 |
| *Anthracocystis walkeri* | KVU 975 | JN367297 | JN367322 |
| *Aureobasidium acericola* | CDH 2020−10 | MT863788 | MT863787 |
| *Aureobasidium aerium* | CFCC 50324 | ON007058 | ON007081 |
| *Aureobasidium bupleuri* | CBS 131304 | JN886792 | JN886792 |
| *Aureobasidium castanea* | CFCC 54591 | MW364284 | MW364275 |
| *Aureobasidium caulivorum* | CBS 242.64 | FJ150871 | FJ150944 |
| *Aureobasidium faidherbiae* | CBS 149677 | OQ628485 | OQ629067 |
| *Aureobasidium harposporum* | CBS 122914 | KT693741 |  |
| *Aureobasidium insectorum* | KCL139 | OP856707 | OP857208 |
| *Aureobasidium intercalariosporum* | MQL9−100 | OP856703 | OP857205 |
| *Aureobasidium iranianum* | CCTU 268 | JX205092 | KM093739 |
| *Aureobasidium khasianum* | NFCCI 4275 | MH188305 | MH188306 |
| *Aureobasidium leucospermi* | CBS 130593 | JN712489 | MH877257 |
| *Aureobasidium lini* | CBS 125.21T | FJ150897 | FJ150946 |
| *Aureobasidium mangrovei* | IBRCM 30265T | KY089085 | KY089084 |
| *Aureobasidium melanogenum* | CBS 105.22 | MH854707 | FJ150926 |
| *Aureobasidium microstictum* | CBS 342.66 | KT693743 | FJ150945 |
| *Aureobasidium microtermitis* | GTS2.7 | MW276135 | MW276136 |
| *Aureobasidium motuoense* | XZY411−4 | OP856710 | OP857211 |
| *Aureobasidium namibiae* | CBS 147.97 | FJ150875 | FJ150937 |
| *Aureobasidium pini* | CFCC 52778 | MK184533 | MK184535 |
| *Aureobasidium planticola* | MDSC−10 | OP856711 | OP857212 |
| *Aureobasidium proteae* | CBS 114273 | JN712491 | JN712557 |
| *Aureobasidium pullulans* | CBS 584.75 | FJ150906 | FJ150942 |
| *Aureobasidium subglaciale* | EXF−2481 | FJ150895 | FJ150913 |
| *Aureobasidium thailandense* | NRRL 58539 | JX462674 | JX462674 |
| *Aureobasidium tremulum* | UN 1 | MK503657 | MK503660 |
| *Aureobasidium welwitschiae* | CBS 149676 | OQ628484 | OQ629066 |
| *Aureobasidium xishuangbannaense* | KUMCC 21-0703 | ON426835 | OP363258 |
| *Aureobasidium zeae* | CBS 265.32 | MH855318 | MH866771 |
| *Babjeviella inositovora* | NRRL Y-12698 | AB054113 | NG_042644 |
| *Barnettozyma californica* | CBS 252 | KY101725 | KY106168 |
| *Barnettozyma hawaiiensis* | CBS 8760 | KY101728 | KY106181 |
| *Barnettozyma populi* | CBS 8094 | KY101730 | EF550277 |
| *Barnettozyma siamensis* | CBS 13392 | KJ413944 | AB741519 |
| *Baueromyces planticola* | CGMCC2.4532 | MN901712 | MN901712 |
| *Blastobotrys indianensis* | CBS 9600 | OL772662 | DQ442690 |
| *Candida aaseri* | CBS 1913 | AY821838 | U45802 |
| *Candida amphicis* | CBS 9877 | EU491501 | AY520327 |
| *Candida andamanensis* | CBS 10859 | AB525239 | AB334210 |
| *Candida anglica* | CBS 4262 | MK394106 | AF245403 |
| *Candida arabinofermentans* | CBS 8468 | KY101943 | KY106302 |
| *Candida atlantica* | CBS 5263 | AJ539368 | U45799 |
| *Candida atmosphaerica* | CBS 4547 | AJ539369 | U45779 |
| *Candida blattariae* | CBS 9876 | FJ715435 | AY640213 |
| *Candida boidinii* | CBS 2428/NRRL Y-2332 | KY101990 | EU011598 |
| *Candida boleticola* | CBS 6420/NRRL Y-17080 | AY821840 | U45777 |
| *Candida buinensis* | CBS 6796 | HQ283376 | U45778 |
| *Candida cerambycidarum* | CBS 9879 | AY964669 | AY520299 |
| *Candida conglobata* | CBS 2018 | AJ539370 | U45789 |
| *Candida dendronema* | CBS 6270 | HQ283365 | U45751 |
| *Candida diddensiae* | CBS 2214 | AY580315 | U45750 |
| *Candida diospyri* | CBS 9769 | AY450919 | AY450918 |
| *Candida endomychidarum* | CBS 9881 | AY964672 | AY520330 |
| *Candida friedrichii* | CBS 4114 | HQ283377 | U45781 |
| *Candida germanica* | CBS 4105 | HQ283366 | AF245401 |
| *Candida gorgasii* | CBS 9880 | AY964670 | AY520300 |
| *Candida insectorum* | CBS 6213 | HQ283372 | U45791 |
| *Candida jaroonii* | CBS 10790 | AB360437 | DQ404493 |
| *Candida kanchanaburiensis* | CBS 11266 | HQ283367 | KY106534 |
| *Candida keroseneae* | CECT 13058 | FJ235128 | FJ357698 |
| *Candida khao-thaluensis* | CBS 8535 | HQ283374 | HQ283383 |
| *Candida koratica* | CBS 10789 | AB360443 | AB354232 |
| *Candida krabiensis* | ATCC MYA-4469 | FJ196784 | FJ196736 |
| *Candida lessepsii* | CBS 9941 | AY964671 | AY640214 |
| *Candida maris* | CBS 5151/NRRL Y-6696 | KY102191 | EU011613 |
| *Candida membranifaciens* | CBS 1952 | AJ606465 | U45792 |
| *Candida methanosorbosa* | CBS 7029/NRRL Y-17320 | KY102211 | EU011600 |
| *Candida michaelii* | CBS 9878 | AY964673 | AY520329 |
| *Candida naeodendra* | CBS 6032 | AY580316 | U45759 |
| *Candida nanaspora* | CBS 7200 | KY102225 | KY106595 |
| *Candida nemodendra* | CBS 6280/NRRL Y-7779 | KY102228 | EU011629 |
| *Candida nitratophila* | CBS 2027/NRRL YB-3654 | JX965188 | EU011606 |
| *Candida oceani* | CBS 11857 | LN870268 | GU002284 |
| *Candida oleophila* | CBS 2219 | KY102254 | KY106621 |
| *Candida ortonii* | CBS 8843 | KY102275 | KY106637 |
| *Candida ovalis* | CBS 7298 | KY102280 | KY106642 |
| *Candida piceae* | CBS 8701/NRRL YB-2107 | KY102332 | EU011633 |
| *Candida pini* | CBS 970/NRRL Y-2023 | KY102335 | EU011639 |
| *Candida pseudoaaseri* | CBS 11170 | JN241686 | JN241689 |
| *Candida railenensis* | CBS 8164 | EU343803 | KY106716 |
| *Candida rishiriensis* | CBS 11662 | KY102366 | KY106727 |
| *Candida santamariae* | CBS 4515 | KY102387 | KY106747 |
| *Candida schatavii* | CBS 6452/NRRL Y-17078 | AY569006 | U45795 |
| *Candida sinolaborantium* | CBS 9940 | FJ172253 | FJ614676 |
| *Candida sithepensis* | ATCC MYA-4470 | FJ196785 | FJ196737 |
| *Candida songkhlaensis* | CBS 10791 | AB360438 | DQ404499 |
| *Candida sonorensis* | CBS 6792 | KY102404 | KY106770 |
| *Candida spencermartinsiae* | CBS 10894 | FJ008050 | FJ008044 |
| *Candida succiphila* | CBS 8003/NRRL Y-11998 | KY102421 | EU011597 |
| *Candida suzukii* | CBS 9253/NRRL Y-27593 | JX965187 | EU011610 |
| *Candida tallmaniae* | CBS 8575 | HQ283378 | HQ283385 |
| *Candida tammaniensis* | CBS 8504 | HQ283375 | AF017243 |
| *Candida taylorii* | CBS 8508 | FJ008051 | FJ008045 |
| *Candida temnochilae* | CBS 9938 | AY964678 | AY242344 |
| *Candida trypodendroni* | CBS 8505 | FJ153212 | AF017240 |
| *Candida vaughaniae* | CBS 8583 | HQ283364 | HQ283381 |
| *Candida vrieseae* | CBS 10829 | FJ755905 | EU200785 |
| *Candida xyloterini* | ATCC 62898 | FJ381696 | FJ381703 |
| *Candida zeylanoides* | CBS 619 | AY542871 | KY106903 |
| *Cephaloascus albidus* | CBS 38977/NRRL Y-7343 | MH861079 | U39474 |
| *Cintractia axicola* | MP 3490 | DQ631908 | DQ631906 |
| *Cryptococcus amylolentus* | CBS 6039 | FJ534872 | FJ534902 |
| *Curvibasidium cygneicollum* | CBS 4551 | AF444490 | AF189928 |
| *Curvibasidium nothofagi* | CBS 8166 | AF444537 | EF551316 |
| *Curvibasidium pallidicorallinum* | CBS 9091 | KY102982 | KY107299 |
| *Curvibasidium rogersii* | NRRL Y-48849 | JX188232 | JX188232 |
| *Cyberlindnera americana* | CBS 5644/NRRL Y-2156 | KY103037 | EF550328 |
| *Cyberlindnera amylophila* | CBS 7020/NRRL YB-1287 | KY103039 | EF550319 |
| *Cyberlindnera bimundalis* | CBS 5642/NRRL Y-5343 | KY103040 | EF550329 |
| *Cyberlindnera culbertsonii* | yHAB218 | KM384448 | KM408121 |
| *Cyberlindnera dasilvae* | UFMG-CM-Y519 | MT312230 | MT311982 |
| *Cyberlindnera dauci* | KBP Y-6686 | MT636878 | MT636878 |
| *Cyberlindnera euphorbiae* | CBS 8033/NRRL Y-17232 | KY103041 | EF550326 |
| *Cyberlindnera euphorbiiphila* | NRRL Y-12742 |  | EF550312 |
| *Cyberlindnera fabianii* | CBS 5640/NRRL 1871 | KY103043 | EF550321 |
| *Cyberlindnera galapagoensis* | UFMGCLQCA-24SC-025 | KJ020281 | KJ020281 |
| *Cyberlindnera jadinii* | CBS 1600 | DQ249199 | AY497671 |
| *Cyberlindnera japonica* | CBS 7209/NRRL YB-2750 | KY103061 | EF550323 |
| *Cyberlindnera lachancei* | CBS 8557/NRRL Y-27008 | KY103063 | EF550313 |
| *Cyberlindnera maclurae* | CBS 8671/NRRL Y-5377 | KY103065 | EF550310 |
| *Cyberlindnera maritima* | CBS 5107 | KY102196 | KY106562 |
| *Cyberlindnera meyerae* | CBS 7076/NRRL Y-17236 | KY103066 | EF550327 |
| *Cyberlindnera mississippiensis* | CBS 7023/NRRL YB-1294 | KY103068 | EF550320 |
| *Cyberlindnera misumaiensis* | CBS 8062/NRRL Y-17389 | KY103070 | EF550306 |
| *Cyberlindnera mrakii* | CBS 1707/NRRL Y-1364 | EU307973 | EF550317 |
| *Cyberlindnera mycetangii* | CBS 8675/NRRL Y-6843 | KY102221 | EF550330 |
| *Cyberlindnera nakhonratchasimensis* | CBS 11706 | KY102223 | KY106593 |
| *Cyberlindnera petersonii* | CBS 5555/NRRL YB-3808 | KY103077 | EF550311 |
| *Cyberlindnera rhizosphaerae* | CBS 11400 | KY103078 | KY107386 |
| *Cyberlindnera rhodanensis* | NRRL Y-7854 |  | EF550325 |
| *Cyberlindnera samutprakarnensis* | CBS 12528 | AB695388 | KY107391 |
| *Cyberlindnera sargentensis* | CBS 6342 | KY103085 | KY107392 |
| *Cyberlindnera saturnus* | CBS 254 | KY103100 | KY107399 |
| *Cyberlindnera suaveolens* | CBS 255 | KY103113 | KY107409 |
| *Cyberlindnera subsufficiens* | CBS 5763 | KY103114 | KY107420 |
| *Cyberlindnera sylvatica* | CBS 16335 |  | MT316316 |
| *Cyberlindnera tropicalis* | DMKU WBBC14 | KY010353 | LC054963 |
| *Cyberlindnera veronae* | CBS 6591 | KY103115 | KY107421 |
| *Cyberlindnera wuzhiensis* | AS2.3480 | FJ606824 | FJ606825 |
| *Cyberlindnera xishuangbannaensis* | NYNU 16752 | KY213821 | KY213813 |
| *Cyberlindnera xylebori* | CBS 12187 | KY103116 | KY107422 |
| *Cyberlindnera xylosilytica* | NRRL YB-2097 | KP232976 | EF550324 |
| *Debaryomyces hansenii* | CBS 767 | JN942652 | JQ689041 |
| *Diddensiella caesifluorescens* | CBS 12613 |  | GU195654 |
| *Dimennazyma cistialbidi* | CBS 10049 | KF036589 | AY562135 |
| *Dothiora viticola* | L9D-17 | KP641179 | KF201298 |
| *Farysia acheniorum* | AS 2.3198 | AB038128 | AF190001 |
| *Farysia itapuensis* | CBS 10428 | DQ767831 | DQ767831 |
| *Farysia setubalensis* | CBS 10241 | EU002888 | EU002857 |
| *Fibulobasidium inconspicuum* | CBS 8237 | AF444318 | AF363641 |
| *Fibulobasidium murrhardtense* | CBS 9109 | GU327540 | KY107700 |
| *Fibulobasidium sirobasidioides* | RJB 12787 |  | AF416644 |
| *Genolevuria amylolytica* | PYCC 5850 | KF036585 | AY562134 |
| *Genolevuria armeniacus* | CBS 10050 | KF036587 | AY562140 |
| *Jaminaea angkorensis* | CBS 10918 | KY103614 | KY107895 |
| *Jaminaea lanaiensis* | CBS 10858 | KY105576 | KY109812 |
| *Jaminaea lantanae* | CGMCC2.3529 | MN901709 | MN901709 |
| *Jaminaea pallidilutea* | IBRC-M 30284 | KY446898 | KY446897 |
| *Jaminaea rosea* | MCA5214 | KR912071 | KR912073 |
| *Kabatiella lini* | CBS 125.21 | FJ150897 | FJ150946 |
| *Kuraishia floccosa* | CBS 10307/NRRL Y-27951 | KY103913 | KM065899 |
| *Kurtzmaniella cleridarum* | CBS 8793 | AY344065 | JQ689038 |
| *Kurtzmaniella fragi* | CBS 7702/NRRL Y-17910 | EU343804 | U71071 |
| *Kurtzmaniella hittingeri* | CBS 13469 | KF582611 | KF582609 |
| *Kurtzmaniella quercitrusa* | CBS 4412/NRRL Y-5392 | AM158924 | KM065904 |
| *Kwoniella bestiolae* | CBS 10118 | FJ534873 | FJ534903 |
| *Kwoniella botswanensis* | CBS 12716 | HF545756 | HF545769 |
| *Kwoniella dejecticola* | CBS 10117 | FJ534874 | FJ534904 |
| *Kwoniella dendrophila* | CBS 6074 | AF444443 | KY108198 |
| *Kwoniella endophytica* | CBS 15359 | MH237945 | MH237945 |
| *Kwoniella europaea* | CBS 7868 | HE984341 | HE996972 |
| *Kwoniella fici* | DBVPG 10122 | MK070336 | MK070318 |
| *Kwoniella heveanensis* | CBS 569 | AF444301 | KY108201 |
| *Kwoniella mangrovensis* | CBS 8507 | AF444646 | AF444742 |
| *Kwoniella newhampshirensis* | CBS 13917/NRRL Y-63731 | KM384101 | KM408127 |
| *Kwoniella ovata* | CGMCC 2.3439 | MK050289 | MK050289 |
| *Kwoniella pini* | VKM Y-2958 | EF672246 | EF672245 |
| *Kwoniella shandongensis* | CBS 12478 | JN160602 | JN160602 |
| *Kwoniella shivajii* | CBS 11374 | KY103937 | KY108204 |
| *Langdonia aristidae* | HUV 19145 | JN367292 | JN367317 |
| *Leucosporidium scottii* | CBS 5930 | AF444495 | AF070419 |
| *Limtongozyma cylindracea* | CBS 6330 | KY102038 | KY106404 |
| *Lodderomyces elongisporus* | ATCC 11503 | GU319980 | HQ876050 |
| *Macalpinomyces eriachnes* | CBS 131454 | JN367287 | JN367312 |
| *Macalpinomyces spermophorus* | JCM 10324/CBS 6389 | AB089372 | AB089373 |
| *Melanopsichium pennsylvanicum* | UMa704 | JN367288 | JN367313 |
| *Meyerozyma amylolytica* | DSM 27310 | KY673531 | KY673531 |
| *Meyerozyma athensensis* | CBS 9840/ATCC MYA-4324 | FJ172250 | AY518528 |
| *Meyerozyma caribbica* | CBS 9966 | KY104222 | KY108516 |
| *Meyerozyma carpophila* | CBS 5256 | MK394110 | KY106386 |
| *Meyerozyma elateridarum* | CBS 9842/ATCC MYA-4325 | FJ196772 | FJ196724 |
| *Meyerozyma guilliermondii* | CBS 2030/NRRL Y-2075 | EF568003 | JQ689047 |
| *Meyerozyma neustonensis* | CBS 11061/SN-92 | EF621563 | EF621559 |
| *Meyerozyma smithsonii* | CBS 9839/ATCC MYA-4323 | FJ172249 | AY518525 |
| *Microbotryum violaceum* | GLM 50283 | DQ640065 | DQ640070 |
| *Moesziomyces antarcticus* | JCM10317 | AB089358 | AB089359 |
| *Moesziomyces bullatus* | JCM 10318 | JN942666 | JN940519 |
| *Moesziomyces parantarcticus* | JCM 11752 | JN942671 | JN940524 |
| *Naematelia aurantia* | CBS 6965 | AF444315 | AF189842 |
| *Naematelia encephala* | CBS 6968 | AF410474 | AF189867 |
| *Neocelosporium eucalypti* | CBS 145086 | MK047452 | MK047502 |
| *Ogataea allantospora* | CBS 10576/NCAIM Y.01822 | KY104390 | EF471446 |
| *Ogataea angusta* | CBS 7073/NRRL Y-2214 | JF756588 | U75524 |
| *Ogataea cecidiorum* | CBS 11522 | KY104392 | KY108670 |
| *Ogataea chonburiensis* | ATCC MYA-4462 | FJ914924 | FJ914941 |
| *Ogataea corticis* | NBRC 1794 | AB440284 | AB440278 |
| *Ogataea deakii* | NCAIM Y.01896 | KC252610 | GQ265921 |
| *Ogataea degrootiae* | CBS 15033 | MG986489 | MG986494 |
| *Ogataea dorogensis* | CBS 9260/NRRL Y-27599 | KY104394 | EU011620 |
| *Ogataea falcaomoraisii* | CBS 9814/UFMG T-264-1 | KY104395 | AY561260 |
| *Ogataea ganodermae* | CBS 10646 | AB440281 | KY108673 |
| *Ogataea glucozyma* | CBS 5766/NRRL YB-2185 | KY104397 | EU011626 |
| *Ogataea haglerorum* | VKPM Y-2583/CBS 14645 | KY492390 | KY492605 |
| *Ogataea henricii* | CBS 5765/NRRL YB-2194 | KY104399 | EU011625 |
| *Ogataea histrianica* | CBS 12779 | HE799677 | HE799677 |
| *Ogataea kanchanaburiensis* | CBS 12673 | AB734093 | AB734090 |
| *Ogataea kodamae* | CBS 7081/NRRL Y-17234 | KY104400 | EU011616 |
| *Ogataea kolombanensis* | ZIM 2322 | HF559222 | FR690079 |
| *Ogataea mangiferae* | CBS 13492/UFMG-CMY-253 | KF585022 | KF585022 |
| *Ogataea methanolica* | CBS 6515/NRRL Y-7685 | KY104403 | EU011638 |
| *Ogataea methylovora* | CBS 7300/NRRL Y-17250 | KY104408 | EU011611 |
| *Ogataea minuta* | NRRL Y-411 |  | EU011618 |
| *Ogataea naganishii* | CBS 6429/NRRL Y-7654 | KY104410 | EU011601 |
| *Ogataea nakhonphanomensis* | ATCC MYA-4463 | FJ914925 | FJ914942 |
| *Ogataea neixiangensis* | NYNU 16951 | KY213810 | KY213811 |
| *Ogataea neopini* | ATCC 28781 | AB440280 | AB440274 |
| *Ogataea nitratoaversa* | NCAIM Y.01837 | EU327036 | EU327032 |
| *Ogataea nonfermentans* | CBS 5764/NRRL YB-2203 | MF574035 | EU011619 |
| *Ogataea nonmethanolica* | UFMG-CA48.1 | OL598077 | KC832477 |
| *Ogataea paradorogensis* | NBRC 100261 | AB437094 | AB437093 |
| *Ogataea paraovalis* | NYNU 167106 | KY213805 | KY213806 |
| *Ogataea parapolymorpha* | CBS 12304 | KY104415 | KY108689 |
| *Ogataea philodendri* | CBS 6075 | KY104419 | KY108691 |
| *Ogataea phyllophila* | CBS 12095 | KY104421 | KY108694 |
| *Ogataea pignaliae* | CBS 6071/NRRL Y-17664 | KY104422 | EU011640 |
| *Ogataea pilisensis* | NCAIM Y.01509/NRRL Y-27598 | EU327035 | EU011630 |
| *Ogataea pini* | JCM 3655 | AB440282 | AB440275 |
| *Ogataea polymorpha* | ATCC 34438 | FJ914915 | FJ914932 |
| *Ogataea populi-albae* | CBS 11363 | KY104440 | KY108712 |
| *Ogataea ramenticola* | CBS 8699/NRRL YB-1985 | KY104442 | EU011608 |
| *Ogataea salicorniae* | CBS 8071 | KY104443 | KY108715 |
| *Ogataea saltuana* | NRRL YB-2437 | JF430479 | EU011627 |
| *Ogataea siamensis* | ATCC MYA-4464 | FJ914926 | FJ914943 |
| *Ogataea thermomethanolica* | CBS 10098 | KY104445 | KY108717 |
| *Ogataea trehaloabstinens* | CBS 9256 | KY104446 | KY108718 |
| *Ogataea trehalophila* | CBS 5361 | KY104447 | KY108719 |
| *Ogataea uvarum* | clone 130 | KY971684 | KY971684 |
| *Ogataea wangdongensis* | CBS 12674 | AB734094 | AB734091 |
| *Ogataea wickerhamii* | CBS 4307/NRRL YB-4943 | JX546582 | EU011612 |
| *Ogataea zsoltii* | NBRC 101079 | AB440285 | AB440279 |
| *Parajaminaea phylloscopi* | CBS 14087 | KF916685 | KF916680 |
| *Phaeotremella pseudofoliacea* | CBS 6969 | AF444431 | AF189868 |
| *Phaffomyces antillensis* | NRRL Y-12881 |  | EU011660 |
| *Phaffomyces opuntiae* | NRRL Y-11707 |  | EF550263 |
| *Phaffomyces thermotolerans* | CBS 7012 | KY104502 | EF550264 |
| *Phaffomyces usticensis* | PYCC 6346 |  | KF719195 |
| *Priceomyces carsonii* | CBS 2285/NRRL YB-4275 | AJ853767 | U45743 |
| *Pseudoseptoria collariana* | CBS 135104 | KF251218 | KF251721 |
| *Pseudosydowia backhousiae* | BRIP 28243 | MW443073 |  |
| *Pseudosydowia eucalypti* | CBS 131832 | MH865934 | MH877368 |
| *Pseudosydowia eucalyptorum* | CBS 145546 | MK876406 | MK876447 |
| *Pseudosydowia indooroopillyensis* | BRIP 28248 | MW443076 | MW443081 |
| *Pseudosydowia louisecottisiae* | BRIP 28159 | MW443071 | MW443079 |
| *Pseudosydowia phantasmae* | CBS 146830 | MW175364 | MW175404 |
| *Pseudosydowia queenslandica* | BRIP 28249 | MW443077 | MW443082 |
| *Pseudotremella moriformis* | CBS 7810 | AF444331 | AF075493 |
| *Pseudotremella nivalis* | CBS 8487 | AF042414 | AF042232 |
| *Pseudozyma alboarmeniaca* | DMST 17135 | AB117961 | AB117961 |
| *Pseudozyma egyptica* | Y4-09 | AB500692 | AB447396 |
| *Pseudozyma henbania* | Y3-09 | AB500691 | AB385596 |
| *Pseudozyma hubeiensis* | CGMCC 2.2493 | DQ008954 | KY108956 |
| *Pseudozyma japonica* | Y7-09 | AB500693 | AB385599 |
| *Pseudozyma mutica* | Y2-09 | AB500690 | AB385595 |
| *Pseudozyma pruni* | CBS 10937 | KY104688 | KY108957 |
| *Pseudozyma thailandica* | CBS 10006 | KY104689 | KY108958 |
| *Quambalaria cyanescens* | CBS 357.73 | DQ119135 | DQ317615 |
| *Rhodosporidiobolus azoricus* | CBS 8949/IGC 5062 | KY104692 | AF321977 |
| *Rhodosporidiobolus colostri* | CBS 348 | KY104695 | AY372177 |
| *Rhodosporidiobolus fluvialis* | CBS 6568 | AY015432 | AF070422 |
| *Rhodosporidiobolus nylandii* | CBS 9093 | KY104699 | KY108966 |
| *Rhodosporidiobolus poonsookiae* | JCM 10207/CBS 9095 | AB030327 | KY108972 |
| *Rhodotorula alborubescens* | JCM 5352/CBS 482 | AB030342 | AF207886 |
| *Rhodotorula araucariae* | CBS 6031 | AF444510 | KY108982 |
| *Rhodotorula babjevae* | CBS 7808 | AF444542 | AF070420 |
| *Rhodotorula dairenensis* | CBS 4406 | KY104735 | AF070429 |
| *Rhodotorula diobovata* | CBS 6085 | AF444502 | AF070421 |
| *Rhodotorula evergladensis* | CBS 10880 | FJ008054 | FJ008048 |
| *Rhodotorula frigidialcoholis* | EXF-10854 | MT560678 | MT569976 |
| *Rhodotorula glutinis* | CBS 20 | AF444539 | AY646097 |
| *Rhodotorula graminis* | CBS 2826 | AF444505 | AF070431 |
| *Rhodotorula kratochvilovae* | CBS 7436 | AF444520 | AF071436 |
| *Rhodotorula mucilaginosa* | CBS 316 | AF444541 | AF070432 |
| *Rhodotorula ngohengohe* | ICMP 22106 | KY285005 | KY285006 |
| *Rhodotorula pacifica* | SY-246 | AB193175 | AB193175 |
| *Rhodotorula paludigena* | CBS 6566 | AF444492 | AF363640 |
| *Rhodotorula sampaioana* | CRUB 1124 | MW879331 | EF595748 |
| *Rhodotorula sphaerocarpa* | CBS 5939 | AF444499 | AF070425 |
| *Rhodotorula taiwanensis* | CBS 11729 | KY104910 | KY109163 |
| *Rhodotorula toruloides* | CBS 6016 | KY104925 | KY109167 |
| *Saccharomycopsis capsularis* | CBS 2519/NRRL Y-17639 | KY105250 | KY109482 |
| *Saitozyma flava* | CBS 331 | AF444338 | AF075497 |
| *Saitozyma paraflava* | VKM Y-2923 T | AY395800 | AY395799 |
| *Scheffersomyces stipitis* | CBS 5773/NRRL Y-7124 | HQ652060 | JQ689044 |
| *Schizonella melanogramma* | CBS 174.42 | DQ832212 | DQ832210 |
| *Selenophoma australiensis* | CBS 124776 | GQ303293 | GQ303324 |
| *Sirobasidium apiculatum* | CBS 14977 | LC203425 | LC203426 |
| *Sirobasidium japonicum* | CBS 14979 | LC203420 | LC016573 |
| *Sirobasidium magnum* | CBS 6803 | KY105422 | AF075475 |
| *Spathaspora gorwiae* | UFMG CM-Y312 | KC959474 | KC959938 |
| *Spencermartinsiella europaea* | CBS 11730 |  | GU597325 |
| *Sporisorium reilianum* | CBS 131460 | KF706438 | KF706430 |
| *Sporisorium scitamineum* | CBS 131463 | JN367296 | JN367321 |
| *Sporobolomyces salmonicolor* | CBS 490 | AY015434 | AF070439 |
| *Starmera amethionina* | NRRL Y-10978 |  | EF550282 |
| *Starmera caribaea* | CBS 7692 | KY105539 | KY109776 |
| *Starmera foglemanii* | UFMG-CM-Y6337 | MN990206 | MG807013 |
| *Starmera ilhagrandensis* | UFMG-CM-Y2251 | MH279972 | MH279972 |
| *Stollia bursa* | KVU 844 | JN367291 | JN367316 |
| *Sugiyamaella americana* | CBS 10352 |  | DQ438193 |
| *Suhomyces tanzawaensis* | NRRL Y-17324 | EU343858 | U44811 |
| *Sydowia polyspora* | CBS 116.29 | MH855019 | MH866487 |
| *Sympodiomycopsis europaea* | CGMCC2.3119 | MN901717 | MN901717 |
| *Teunia acericola* | NYNU 2111141 | OM017172 | OM017170 |
| *Teunia betulae* | CBS 13896/NRRL Y-63732 | KM384102 | KM408130 |
| *Teunia cuniculi* | CBS 10309 | KM079158 | KY106982 |
| *Teunia globosa* | CGMCC2.5648 | MK050288 | MK050288 |
| *Teunia helanensis* | CGMCC2.4450 | MK050287 | MK050287 |
| *Teunia korlaensis* | CGMCC2.3835 | MK050286 | MK050286 |
| *Teunia lichenophila* | KBP:Y-6495 | MN128421 | MN128421 |
| *Teunia mussaendrae* | NYNU 23232 | OQ851888 | OQ851887 |
| *Teunia nitrariae* | 17-P246-2-1 | OM417183 | OM417183 |
| *Teunia qingyuanensis* | NYNU 22475 | OP269841 | OP269842 |
| *Teunia rosae* | NBRC 114210 | MK942579 | MK942561 |
| *Teunia rudbeckiae* | NBRC 114212 | MK942577 | MK942559 |
| *Teunia siamensis* | DMKU-XD44 | LC440108 | LC420623 |
| *Teunia tronadorensis* | CRUB 1299 | MF959620 | MF959620 |
| *Teunia turchettiae* | KBP Y-6607 | MT470198 | MT470198 |
| *Teunia virginiahalliae* | BRIP 64084e | OR660683 |  |
| *Tremella diederichiana* | MAF-Lich. 19739 | KT334580 | KT334592 |
| *Tremella endosporogena* | MAF-Lich. 19742 | KT334579 | KT334591 |
| *Tremella mayrhoferi* | AM1068 | MH168093 | MH168097 |
| *Trigonosporomyces hylophilus* | CBS 6226 | KY105766 | KY109977 |
| *Trimorphomyces papilionaceus* | CBS 443.92 | AF444483 | AF075491 |
| *Trimorphomyces sakaeratica* | CBS 9934 | AY217651 | AY211546 |
| *Urocystis colchici* | CBS 283.28 | DQ839596 | DQ838576 |
| *Ustanciosporium gigantosporum* | CBS 131478 | JN367300 | JN367325 |
| *Ustilago abaconensis* | CBS 8380 | FJ008053 | FJ008047 |
| *Ustilago cynodontis* | HRK 040/MS | AY740168 | AY740168 |
| *Ustilago filiformis* | HRK 025 | JN367302 | JN367328 |
| *Ustilago hordei* | CBS 131470 | KF706437 | KF706429 |
| *Ustilago maydis* | CBS 504.76 | AY854090 | AF453938 |
| *Ustilago shanxiensis* | CBS 10075 | KY105788 | KY109996 |
| *Ustilago tritici* | CBS 669.70 | DQ846894 | DQ094784 |
| *Ustilago vetiveriae* | HUV 17954 | AY345011 | JN367337 |
| *Ustilago xerochloae* | KVU 1000 | JN367311 | JN367339 |
| *Vishniacozyma alagoana* | BRT 179 | MH885328 | MH909005 |
| *Vishniacozyma carnescens* | CBS 973 | KF036588 | AB035054 |
| *Vishniacozyma dimennae* | CBS 5770 | AF410473 | AF075489 |
| *Wickerhamomyces anomalus* | CBS 5759/NRRL Y-366 | DQ249196 | EF550341 |
| *Wickerhamomyces canadensis* | NRRL Y-1888 |  | EF550300 |
| *Wickerhamomyces ciferrii* | NRRL Y-1031 | FJ153218 | EF550339 |
| *Yamadazyma akitaensis* | CBS 6701 | DQ409164 | U45766 |
| *Yamadazyma barbieri* | CBS 14301 | LT547714 | LT547716 |
| *Yamadazyma cocois* | VTCC 920004 | MN764369 | MN764369 |
| *Yamadazyma dushanensis* | CBS 13914 | KM272249 | KM272248 |
| *Yamadazyma endophytica* | CBS 14163 | KT307981 | KT307981 |
| *Yamadazyma epiphylla* | CBS 13384 | LC006082 | LC006026 |
| *Yamadazyma insecticola* | CBS 13382 | LC006081 | DQ400379 |
| *Yamadazyma kitorensis* | CBS 14158 | LC060995 | LC060995 |
| *Yamadazyma laniorum* | CBS 14780 | KY588337 | KY588136 |
| *Yamadazyma luoyangensis* | NYNU 201023 | MW365549 | MW365545 |
| *Yamadazyma mexicana* | CBS 7066 | AB054110 | U45797 |
| *Yamadazyma nakazawae* | CBS 6700 | EU343867 | U45748 |
| *Yamadazyma olivae* | CBS 11171 | FJ715432 | FJ715430 |
| *Yamadazyma ovata* | NYUN 191125 | MT990560 | MT990559 |
| *Yamadazyma paraaseri* | NYNU 1811114 | MK682794 | MK682805 |
| *Yamadazyma paraphyllophila* | CBS 9928 | AY559447 | AY562397 |
| *Yamadazyma philogaea* | CBS 6696 | AB054107 | U45765 |
| *Yamadazyma phyllophila* | CBS 12572 | AB734050 | AB734047 |
| *Yamadazyma riverae* | CBS 14121 | KP900044 | KP900043 |
| *Yamadazyma scolyti* | CBS 4802 | EU343807 | U45788 |
| *Yamadazyma siamensis* | CBS 12573 | AB734049 | AB734046 |
| *Yamadazyma takamatsuzukensis* | CBS 10916 | AB365470 | AB365470 |
| *Yamadazyma tenuis* | CBS 615 | HQ283371 | U45774 |
| *Yamadazyma terventina* | CBS 12510 | JQ247717 | JQ247717 |
| *Yamadazyma triangularis* | CBS 4094 | EU343869 | U45796 |
| *Yamadazyma tumulicola* | CBS 10917 | AB365463 | AB365463 |
| *Yamadazyma ubonensis* | CBS 12859 | KY105957 | AB759913 |
| *Yunzhangia sonckii* | CBS 6713 | AF444601 | AF189969 |
| *Zygoascus biomembranicola* | CBS 14157 |  | LC060997 |
| *Zygoascus bituminiphilus* | CBS 8813 |  | AF294910 |
| *Zygoascus detingensis* | NYNU 201087 |  | MW368733 |
| *Zygoascus flipseniorum* | CBS 14876 |  | MF695078 |
| *Zygoascus hellenicus* | CBS 5839 |  | DQ438216 |
| *Zygoascus meyerae* | CBS 4099 |  | DQ438189 |
| *Zygoascus ofunaensis* | CBS 8129 |  | DQ438192 |
| *Zygoascus polysorbophila* | CBS 7317 |  | DQ438188 |
| *Zygoascus tannicola* | CBS 6065 |  | KY110232 |

**Table S3.** Yeasts used in fruit extract assimilation assays.

| **Strain ID** | **LESF ID** | **Species** | **Strain ID** | **LESF ID** | **Species** |
| --- | --- | --- | --- | --- | --- |
| B24 | LESF 1544 | *Cyberlindnera* sp. | B376 | LESF 1525 | *Ogataea* sp. |
| B56 | LESF 1556 | *Cyberlindnera* sp. | B377 | LESF 1533 | *Cyberlindnera* sp. |
| B62 | LESF 1542 | *Cyberlindnera* sp. | B379 | LESF 1540 | *Ogataea* sp. |
| B74 | LESF 1515 | *Cyberlindnera* sp. | B380 | LESF 1547 | *Cyberlindnera* sp. |
| B81 | LESF 1526 | *Cyberlindnera* sp. | B393 | LESF 1557 | *Cyberlindnera* sp. |
| B95 | LESF 1509 | *Cyberlindnera* sp. | B417 | LESF 1546 | *Cyberlindnera* sp. |
| B100 | LESF 1552 | *Cyberlindnera* sp. | B418 | LESF 1511 | *Yamadazyma riverae* |
| B101 | LESF 1543 | *Cyberlindnera* sp. | B450 | LESF 1539 | *Cyberlindnera* sp. |
| B116 | LESF 1522 | *Cyberlindnera* sp. | B461 | LESF 1305 | *Cyberlindnera* sp. |
| B117 | LESF 1534 | *Cyberlindnera* sp. | B467 | LESF 1532 | *Cyberlindnera* sp. |
| B122 | LESF 1530 | *Cyberlindnera* sp. | B469 | LESF 1529 | *Cyberlindnera* sp. |
| B127 | LESF 1535 | *Ogataea* sp. | B477 | LESF 1510 | *Zygoascus ofunaensis* |
| B130 | LESF 1523 | *Cyberlindnera* sp. | B483 | LESF 1514 | *Cyberlindnera* sp. |
| B131 | LESF 1553 | *Cyberlindnera* sp. | B489 | LESF 1536 | *Cyberlindnera* sp. |
| B274 | LESF 1519 | *Cyberlindnera* sp. | B523 | LESF 1524 | *Cyberlindnera* sp. |
| B307 | LESF 1548 | *Cyberlindnera* sp. | B545 | LESF 1545 | *Meyerozyma caribbica* |
| B310 | LESF 1513 | *Cyberlindnera* sp. | B557 | LESF 1517 | *Yamadazyma riverae* |
| B312 | LESF 1549 | *Cyberlindnera* sp. | B560 | LESF 1516 | *Yamadazyma riverae* |
| B320 | LESF 1550 | *Cyberlindnera* sp. | B563 | LESF 1512 | *Meyerozyma caribbica* |
| B328 | LESF 1541 | *Yamadazyma riverae* | B565 | LESF 1537 | *Meyerozyma caribbica* |
| B343 | LESF 1538 | *Yamadazyma riverae* | B592 | LESF 1518 | *Yamadazyma riverae* |
| B345 | LESF 1432 | *Cyberlindnera* sp. | B599 | LESF 1531 | *Yamadazyma riverae* |
| B350 | LESF 1528 | *Cyberlindnera* sp. | B612 | LESF 1520 | *Meyerozyma caribbica* |
| B365 | LESF 1527 | *Ogataea* sp. | B614 | LESF 1469 | *Meyerozyma caribbica* |

**Table S4.** Species diversity estimators from infested fruits samples.

| **Estimator** | **Value** | **Estimator** | **Value** |
| --- | --- | --- | --- |
| Samples | 31 | Jackknife 1 | 19.74 |
| Richness | 12 | Jackknife 2 | 26.32 |
| Abundance | 598 | Chao - 1 | 14.99 |
| Shannon | 0.88 | Chao - 2 | 42.97 |
| Simpson | 0.36 | Bootstrap | 15.11 |
| Inverse Simpson | 1.56 | ACE | 18.24 |
| Jevennes | 0.35 | ICE | 27.38 |
| Eevenness | 0.20 |  |  |

**Table S5.** Preference values (absolute number) of females and males in each of the assessments. Being, Cy – *Cyberlindnera* sp. B56 (in fermented fruit extract), Y – *Yamadazyma riverae* B343 (in fermented fruit extract), M – *Meyerozyma caribbica* B565 (in fermented fruit extract), E – Fruit extract, C – Sterile water, N – Absence of choose, CE – Cells of *Cyberlindnera* sp. B56 recovered and suspended in PBS 1×, Cy/E – Fermented fruit extract containing *Cyberlindnera* sp. B56 and F/E – Filtered sterilized fermented fruit extract.

|  | **Assay - 1** | | | | | |
| --- | --- | --- | --- | --- | --- | --- |
|  | **Cy** | **Y** | **E** | **C** | **N** |  |
| **Female** | 23 | 7 | 3 | 0 | 2 |  |
| **Male** | 13 | 3 | 4 | 0 | 0 |  |
|  |  |  |  |  |  |  |
|  | **Assay - 2** | | | | | |
|  | **Cy** | **M** | **E** | **C** | **N** |  |
| **Female** | 24 | 7 | 3 | 0 | 1 |  |
| **Male** | 11 | 6 | 2 | 0 | 1 |  |
|  |  |  |  |  |  |  |
|  | **Assay - 3** | | | | | |
|  | **Y** | **M** | **E** | **C** | **N** |  |
| **Female** | 9 | 10 | 8 | 2 | 6 |  |
| **Male** | 2 | 9 | 2 | 0 | 7 |  |
|  |  |  |  |  |  |  |
|  | **Assay - 4** | | | | | |
|  |  |  |  |  |  |  |
|  | **C** | **CE** | **Cy/E** | **F/E** | **E** | **N** |
| **Female** | 6 | 5 | 18 | 31 | 9 | 3 |
| **Male** | 2 | 1 | 7 | 8 | 6 | 0 |

**Table S6.** Result of the Chi-Square Goodness of Fit test comparing the beetle choices in the assays with the expected distribution of a stochastic probability.

| **Assay** | **X-squared** | **gl** | **P** |
| --- | --- | --- | --- |
| 1 – Females | 49.429 | 4 | 4.75E-10 |
| 1 – Males | 28.5 | 4 | 9.88E-06 |
| 2 – Females | 55.714 | 4 | 2.30E-11 |
| 2 – Males | 20.5 | 4 | 0.001 |
| 3 – Females | 5.714 | 4 | 0.222 |
| 3 – Males | 14.5 | 4 | 0.006 |
| 4 – Females | 47.667 | 5 | 4.15E-09 |
| 4 – Males | 14.5 | 5 | 0.013 |

**Table S7.** Result of the binomial exact test performed as post-hoc to compare the observed preferences with the expected stochastic distribution of each choice. P values are presented by sex and assays, being: Cy – *Cyberlindnera* sp. B56 (in fermented fruit extract), Y – *Yamadazyma riverae* B343 (in fermented fruit extract), M – *Meyerozyma caribbica* B565 (in fermented fruit extract), E – Fruit extract, C – Sterile water, N – Absence of choose, CE – Cells of *Cyberlindnera* sp. B56 recovered and suspended in PBS 1×, Cy/E – Fermented fruit extract containing *Cyberlindnera* sp. B56 and F/E – Filtered sterilized fermented fruit extract.

| Teste *Exact binomial* | | | | | | |
| --- | --- | --- | --- | --- | --- | --- |
|  | **Assay - 1** | | | | | |
|  | **Cy** | **Y** | **E** | **C** | **N** |  |
| **Female** | 5.49E-09 | 1 | 0.1352 | 0.00093 | 0.03322 |  |
| **Male** | 1.52E-05 | 0.7818 | 1 | 0.02151 | 0.02151 |  |
|  |  |  |  |  |  |  |
|  | **Assay - 2** | | | | | |
|  | **Cy** | **M** | **E** | **C** | **N** |  |
| **Female** | 6.74E-10 | 1 | 0.1352 | 0.00093 | 0.00921 |  |
| **Male** | 0.00056 | 0.265 | 0.4019 | 0.02151 | 0.1559 |  |
|  |  |  |  |  |  |  |
|  | **Assay - 3** | | | | | |
|  | **Y** | **M** | **E** | **C** | **N** |  |
| **Female^1^** | - | - | - | - | - |  |
| **Male** | 0.4019 | 0.00998 | 0.4019 | 0.02151 | 0.09822 |  |
|  |  |  |  |  |  |  |
|  | **Assay - 4** | | | | | |
|  |  |  |  |  |  |  |
|  | **C** | **CE** | **Cy/E** | **F/E** | **E** | **N** |
| **Female** | 0.05787 | 0.02568 | 0.07949 | 1.27E-07 | 0.4292 | 0.00226 |
| **Male** | 0.4114 | 0.1641 | 0.1038 | 0.04802 | 0.2725 | 0.02434 |

^1^ No analysis was carried out for this interaction, since no statistical differences were observed in the Chi-Square Goodness of Fit test (X-squared = 5.714; gl = 4; P = 0.222) comparing the recorded choices with the expected distribution of a stochastic probability.

**Table S8.** Result of the Chi-Square Goodness of Fit test comparing the beetle choices in the assays with the expected distribution of a stochastic probability, excluding the absence of choice as a variable.

| **Assay** | **X-squared** | **gl** | **P** |
| --- | --- | --- | --- |
| 1 – Females | 38.152 | 3 | 2.62E-08 |
| 1 – Males | 18.8 | 3 | 0.001 |
| 2 – Females | 40.588 | 3 | 8.00E-09 |
| 2 – Males | 14.895 | 3 | 0.002 |
| 3 – Females | 5.3448 | 3 | 0.148 |
| 3 – Males | 14.385 | 3 | 0.002 |
| 4 – Females | 34.406 | 4 | 6.15E-07 |
| 4 – Males | 8.0833 | 4 | 0.089 |

**Table S9.** Result of the binomial exact test performed as post-hoc to compare the observed preferences with the expected stochastic distribution of each choice, excluding the absence of choice as a variable. P values are presented by sex and assays, being: Cy – *Cyberlindnera* sp. B56 (in fermented fruit extract), Y – *Yamadazyma riverae* B343 (in fermented fruit extract), M – *Meyerozyma caribbica* B565 (in fermented fruit extract), E – Fruit extract, C – Sterile water, N – Absence of choose, CE – Cells of *Cyberlindnera* sp. B56 recovered and suspended in PBS 1×, Cy/E – Fermented fruit extract containing *Cyberlindnera* sp. B56 and F/E – Filtered sterilized fermented fruit extract.

| Teste *Exact binomial* | | | | | |
| --- | --- | --- | --- | --- | --- |
|  | **Assay - 1** | | | | |
|  | **Cy** | **Y** | **E** | **C** |  |
| **Female** | 4,49E-07 | 0.5642 | 0.02939 | 9.37e-05 |  |
| **Male** | 0.0001837 | 0.4394 | 0.7977 | 0.007113 |  |
|  |  |  |  |  |  |
|  | **Assay - 2** | | | | |
|  | **Cy** | **M** | **E** | **C** |  |
| **Female** | 7.311e-08 | 0.608 | 0.1931 | 9.37e-05 |  |
| **Male** | 0.003942 | 0.265 | 0.4019 | 0.007113 |  |
|  |  |  |  |  |  |
|  | **Assay - 3** | | | | |
|  | **Y** | **M** | **E** | **C** |  |
| **Female^1^** | - | - | - | - |  |
| **Male** | 0.1931 | 0.06524 | 0.1931 | 0.007113 |  |
|  |  |  |  |  |  |
|  | **Assay - 4** | | | | |
|  |  |  |  |  |  |
|  | **C** | **CE** | **Cy/E** | **F/E** | **E** |
| **Female** | 0.01149 | 0.004502 | 0.3018 | 9.399e-06 | 0.1393 |
| **Male^1^** | - | - | - | - | - |

^1^ No analysis was carried out for this interaction, since no statistical differences were observed in the Chi-Square Goodness of Fit test comparing the recorded choices with the expected distribution of a stochastic probability.

**Table S10.** Glm and model fit statistics.

| **Glm Negative Binomial distribution** | | |
| --- | --- | --- |
| **Factor** | **Estimate** | **p-value** |
| Intercept | -8.56 | 0.008 |
| Precipitation | 0.36 | 0.056 |
| Humidity | 2.292 | 2.2E-08 |
| Precipitation:Humidity | -0.005 | 0.069 |
| **Analysis of Deviance** | | |
|  | **Df** | **p-value** |
| Precipitation | 1 | 2.90E-05 |
| Humidity | 1 | 1.71E-06 |
| Precipitation:Humidity | 1 | 0.038 |

**References**

Chao, A., Gotelli, N. J., Hsieh, T. C., Sander, E. L., Ma, K. H., Colwell, R. K., Ellison, A. M. (2014). Rarefaction and extrapolation with Hill numbers: a framework for sampling and estimation in species diversity studies. Ecological monographs, 84(1), 45-67.

Carvalho, P. E. R. Espécies arbóreas brasileiras. Brasília: Embrapa Informação Tecnológica; Colombo: Embrapa Florestas, 2003. v. 1, p. 743-749.

Colwell, R. K. (2013). EstimateS: Statistical estimation of species richness and shared species from samples. Version 9.

Hartig F (2022). DHARMa: Residual Diagnostics for Hierarchical (Multi-Level / Mixed) Regression Models. R package version 0.4.6, https://CRAN.R-project.org/package=DHARMa.

Kindt R, Coe R (2005). Tree diversity analysis. A manual and software for common statistical methods for ecological and biodiversity studies. World Agroforestry Centre (ICRAF). ISBN 92-9059-179-X, http://www.worldagroforestry.org/output/tree-diversity-analysis.

Oksanen J, et al. (2024). vegan: Community Ecology Package. R package version 2.6-6.1, https://CRAN.R-project.org/package=vegan.

T. C. Hsieh, K. H. Ma; Anne Chao. 2024 iNEXT: iNterpolation and EXTrapolation for species diversity. R package version 3.0.1 URL: http://chao.stat.nthu.edu.tw/wordpress/software-download/.

Posit team (2023). RStudio: Integrated Development Environment for R. Posit Software, PBC, Boston, MA. URL http://www.posit.co/.

R Core Team (2023). R: A Language and Environment for Statistical Computing. R Foundation for Statistical Computing, Vienna, Austria. https://www.R-project.org/.
